# Modulation of neural activity in frontopolar cortex drives reward-based motor learning

**DOI:** 10.1038/s41598-021-98571-y

**Published:** 2021-10-13

**Authors:** M. Herrojo Ruiz, T. Maudrich, B. Kalloch, D. Sammler, R. Kenville, A. Villringer, B. Sehm, V. V. Nikulin

**Affiliations:** 1 Psychology Department, Goldsmiths University of London, London, UK; 2 Center for Cognition and Decision Making, National Research University Higher School of Economics, Moscow, Russian Federation; 3 Department of Neurology, Max Planck Institute for Human Cognitive and Brain Sciences, Leipzig, Germany; 4 Research Group Neurocognition of Music and Language, Max Planck Institute for Empirical Aesthetics, Frankfurt am Main, Germany; 5 Department of Neurology, University Hospital Halle (Saale), Halle, Germany

**Keywords:** Neuroscience, Psychology

## Abstract

The frontopolar cortex (FPC) contributes to tracking the reward of alternative choices during decision making, as well as their reliability. Whether this FPC function extends to reward gradients associated with continuous movements during motor learning remains unknown. We used anodal transcranial direct current stimulation (tDCS) over the right FPC to investigate its role in reward-based motor learning. Nineteen healthy human participants practiced novel sequences of finger movements on a digital piano with corresponding auditory feedback. Their aim was to use trialwise reward feedback to discover a hidden performance goal along a continuous dimension: timing. We additionally modulated the contralateral motor cortex (left M1) activity, and included a control sham stimulation. Right FPC-tDCS led to faster learning compared to lM1-tDCS and sham through regulation of motor variability. Bayesian computational modelling revealed that in all stimulation protocols, an increase in the trialwise expectation of reward was followed by greater exploitation, as shown previously. Yet, this association was weaker in lM1-tDCS suggesting a less efficient learning strategy. The effects of frontopolar stimulation were dissociated from those induced by lM1-tDCS and sham, as motor exploration was more sensitive to inferred changes in the reward tendency (volatility). The findings suggest that rFPC-tDCS increases the sensitivity of motor exploration to updates in reward volatility, accelerating reward-based motor learning.

## Introduction

One of the hallmarks of motor skill learning is the reduction in movement variability^1,2^. As the dancer learns to perform pirouettes, the irregularity in the movement decreases and the turns become smoother. In this context, movement variability is regarded as motor noise, and is dissociated from the intentional use of motor variability, termed motor exploration^3,4^. Motor learning can also involve motor exploration, particularly when learning from reinforcement, such as feedback about success or failure^2,4,5^. In this scenario, initial exploration of movement variables in a continuous space is followed by the exploitation of inferred optimal movements and gradually refined by reducing motor noise^6^. How agents decide about motor exploration and exploitation is the subject of an increasing number of studies on motor learning as a decision-making process^4,7–9^. Because motor learning often depends on making the right decisions about a movement, brain regions involved in regulating the exploration–exploitation tradeoff in cognitive or economic decision-making tasks may also modulate the use of variability during motor decision making.

Here, we postulate that the human frontopolar cortex (FPC) may have a crucial role in driving motor decision making when external reward signals are available to drive exploration–exploitation. In decision-making tasks involving multiple choices, the right FPC has been identified as promoting exploration^10,11^, and tracking the reward value associated with competing options, strategies or goals^12^. Furthermore, FPC accelerates the learning of novel rules^13^. Because motor learning takes place in a continuous movement space^7^, we reasoned that the capability of FPC to monitor multiple discrete choices and their reward would make it an ideal candidate to track the reward associated with continuous movement parameters.

To identify the role of the FPC in reward-based motor learning, we used anodal transcranial direct current stimulation (tDCS) over the right FPC while participants completed our recently developed reward-dependent motor sequence learning paradigm^14^. Participants practiced novel sequences of finger movements on a digital piano with corresponding auditory feedback. They were instructed to use trialwise reward feedback to discover a hidden performance goal along a continuous dimension: timing. To discover the hidden performance target, participants had to deviate from an isochronous performance of temporal intervals. Thus, success in this task was coupled to an intentional exploration of anysochronous timing patterns. To assess unintentional motor variability, we took an independent measurement of each participant’s baseline motor noise^4^.

During learning, trial-to-trial exploratory behavioural changes were assessed using a Bayesian computational modelling framework, the Hierarchical Gaussian Filter (HGF^15^). This framework allowed us to estimate how participants adapted their behaviour (regulating motor variability across trials) following updated beliefs about the reward tendency and its rate of change, termed environmental volatility^15^). Beliefs in the HGF are updated trial to trial using prediction errors (PE)—the mismatch between expected and observed states; PEs are modulated by the reliability (or precision) that subjects assign to the state being updated. We hypothesised that FPC-tDCS would modulate behavioural exploration in response to updates in reward and volatility estimates. The *right* FPC was selected over the left FPC due to its greater engagement in regulating the exploration–exploitation balance^11,16^.

As control tDCS condition we modulated motor cortex activity using contralateral (left) M1 tDCS^17,18^. Animal and human neurophysiology studies highlight a crucial role of M1 in modulating motor variability and in processing reward^6,14,19^. Additional evidence comes from transcranial magnetic stimulation (TMS) and tDCS studies^20–24^, with investigations also linking M1 to decision making^25–27^. In our study, active tDCS conditions were contrasted to sham stimulation. The effect of the tDCS stimulation protocols on the individual brain was further assessed using simulations of the electric field strength guided by individual T1-weighted anatomical magnetic resonance images (MRI).

The central hypothesis was that rFPC-tDCS improves learning of the continuous reward landscape associated with movement parameters by balancing the exploration–exploitation tradeoff. Additionally, rFPC-tDCS was hypothesised to modulate task-related timing exploration following updates in beliefs on reward and volatility. On the other hand, animal studies demonstrate a link between variability in motor cortex and behavioural variability^6,19^, with reduced M1 variability contributing to the refinement of task-related motor variability as training progresses^6^. Yet, these studies suggest that the role of M1 in this process may be relevant in the later stages of learning^19^. Accordingly, we hypothesised that the effect of lM1-tDCS on the modulation of intentional motor variability would manifest later during training when compared to rFPC-tDCS.

First, we show that rFPC-tDCS compared to sham and lM1-tDCS accelerated learning from online to offline learning blocks primarily through regulation of task-related motor variability. Second, the Bayesian modelling analysis revealed that rFPC-tDCS and sham promote more exploitative behaviour than lM1-tDCS when the expectation on the reward tendency increases. Moreover, as learning progresses, the reward tendency increases and the environment becomes more stable, we show that frontopolar stimulation is associated with a more pronounced tendency to exploit the inferred timing solution (smaller timing exploration).

## Results

**Figure 1:**
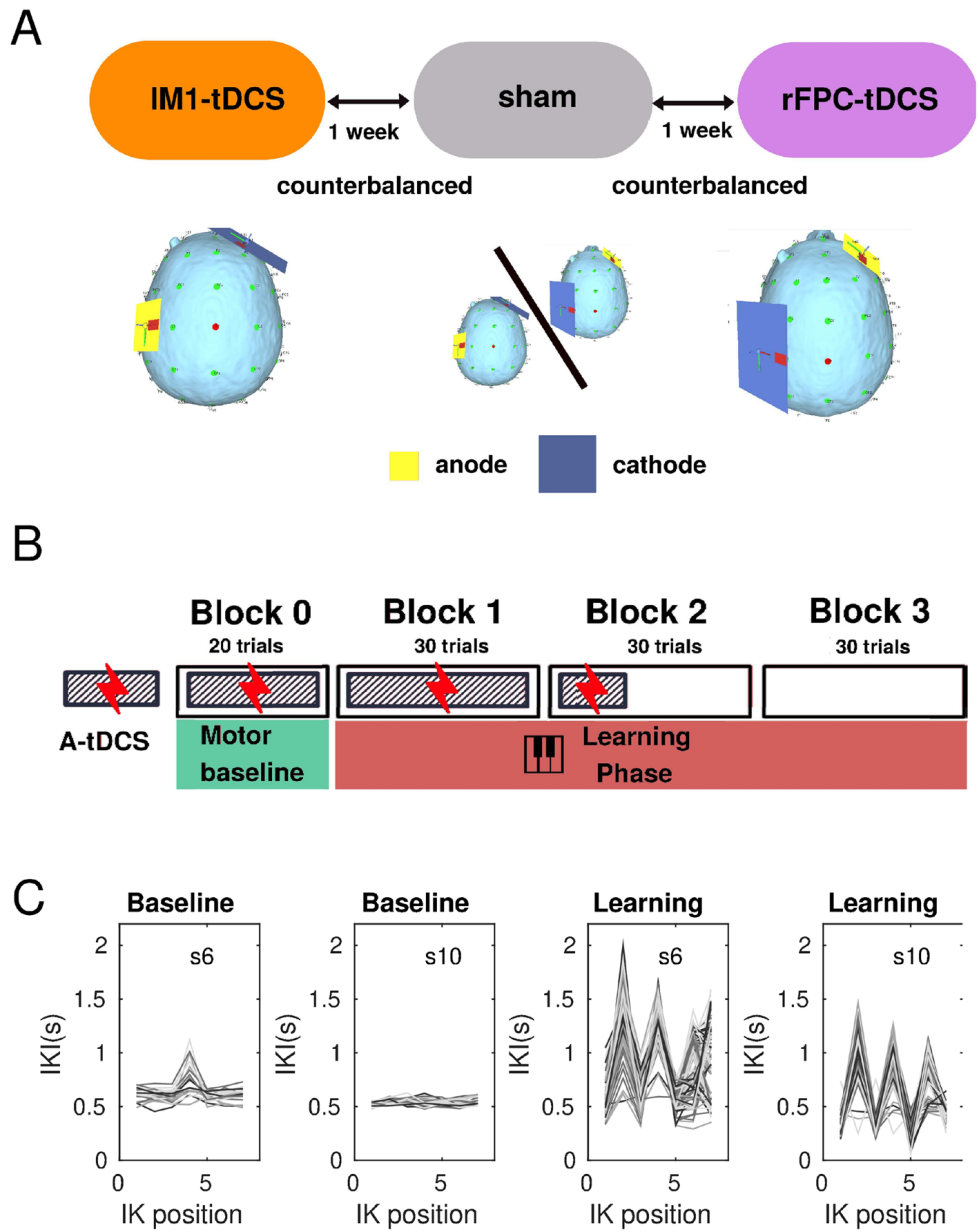
Experimental design. (**A**) All participants were tested on three separate weeks during which either an active tDCS protocol over the lM1, or the rFPC, or a sham stimulation condition were applied. The order of the tDCS sessions was counterbalanced across participants. The position of the anode and cathode for each stimulation condition is illustrated in one participant. The electrode placement during sham was taken from active rFPF-tDCS or lM1-tDCS (equal split across participants). Electrode placement figures were created with the freely available SimNIBS 2.1 software^30,31^, https://simnibs.github.io/simnibs. (**B**) All tDCS protocols extended for 20 min, which included (i) an initial 3 min resting phase, (ii) a baseline phase of regular isochronous motor performance, and (iii) part of the reward-based learning blocks (1 + 1/3 blocks). (**C**) Illustration of timing performance during sham in the baseline and learning phase in two participants. Timing was measured using the inter-keystroke-interval (IKI, s), and shown for each IK position (1–7 for sequences of 8 key presses). Different trajectories denote performance in different trials. During baseline, participants were instructed to keep a regular, isochronous rhythm. During reward-based learning, they had to vary the timing dimension (IKI pattern) to discover the hidden performance target.

Nineteen right-handed participants took part in our study implementing a sham-controlled, double-blinded, cross-over design. They underwent three types of a tDCS protocol (rFPC, lM1, sham condition) over three separate weeks in a pseudo-randomised counterbalanced order across participants (Fig. 1A). Study procedure for each session was identical, with active or sham tDCS applied to the target area for a period of 20 min during task performance (Fig. 1B). One exception was the last block of the learning phase, which was initiated 5 min after the cessation of tDCS and thus served to assess offline effects on learning. Notably, however, anodal tDCS stimulation effects on motor learning have been shown to last for at least 30 min after halting tDCS stimulation^18,28^, but see^29^ showing null results on immediate offline motor effects. Accordingly, we assumed that the recent stimulation would strongly influence performance during the last block ($\sim$ 5 min after tDCS cessation). To account for a possible differential effect of tDCS protocols on the offline (3) and online (1) blocks, we planned to assess the offline minus online contrast across stimulation conditions (see “Materials and methods”).

All participants received rFPC anodal tDCS, lM1 anodal tDCS, and sham tDCS (targetting either rFPC or lM1, 50%–50% split across participants). In each tDCS session, they completed a motor task with their right hand on a digital piano (Yamaha Clavinova CLP-150). The task consisted of an initial baseline phase of 20 trials of regular isochronous performance, followed by three blocks of 30 trials of reward-based sequence learning (Fig. 1B). Each week, participants played a different sequence of key presses and associated auditory feedback on the piano (counterbalanced order; see details in Figure S1 and in “Materials and methods”). The baseline phase allowed us to obtain a measure of motor noise, which represents the residual variability that is expressed when aiming to accurately reproduce the same action^4,32^ (Fig. 1C). During reward-based learning blocks, participants completed an adaptation of our recently developed reward-based motor sequence learning task^14^. In this task, participants receive continuous reward in the form of a trialwise feedback score (range 0–100) to discover a hidden performance goal: a timing pattern. Crucially, participants were explicitly instructed to vary the timing of the performance during the learning phase as this dimension was associated with reward. The use of continuous feedback scores to guide learning was based on previous studies investigating motor variability during reward-based learning^4,5^ and motor decision making^33^. Continuous feedback has been shown to be more informative than binary signals (success/failure), contributing to faster learning^34^.

### Behavioural changes across blocks

Analysis of the behavioural data focused on the assessment of motor variability across trials and along two dimensions: time, which was the *instructed* task-related dimension and measured here using the inter-keystroke-interval (IKI, seconds) index; and keystroke velocity (arbitrary units, a.u.), which was the non-task-related dimension. The keystroke velocity is associated with the loudness of the key press. We used the coefficient of variation (cv = sd/mean) across trials to assess the extent of variability within a block in relation to the mean of the sample. The achieved scores and other general performance variables were also evaluated (“Materials and methods”).

**Figure 2:**
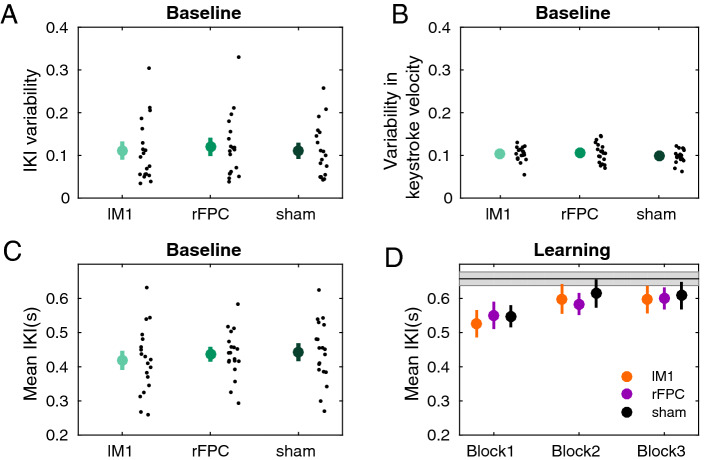
Behavioural results during baseline. (**A–D**) Effects of stimulation conditions on performance variables during the baseline phase. Large coloured dots indicate means, with error bars denoting ± SEM. (**A**) Temporal variability across trials in the baseline block, measured with the coefficient of variation of IKI across trials (cv = std/mean, dimensionless). (**B**) Variability in keystroke velocity or loudness across trials, measured as in (**A**). (**C**) Mean performance tempo, mean IKI (s). (**D**) Average performance tempo during learning. The gray horizontal line indicates the mean tempo of the hidden target solutions.

The different tDCS protocols did not have dissociable effects on baseline motor variability for timing ($P = 0.91$, one-way factorial analysis with synchronised rearrangements; Fig. 2). Neither was there a significant main effect of factor Stimulation on the variability of keystroke velocity at baseline ($P = 0.85$). General performance parameters did not differ in this phase of the experiment as a function of the stimulation protocol either (Supplementary Results), suggesting that any differential stimulation effects on the subsequent learning phases are not modulated by baseline effects.

During reward-based learning participants demonstrated an initial tendency to explore timing solutions that were close-by but also far away in the movement space (Figure S2, S3, see also the right panels on Fig. 1C). This indicated that they initially explored the task-related temporal dimension. Participants explored timing patterns by (i) changing the ratio of IKI values across neighboring keystrokes (different shape of IKI patterns) but also by (ii) increasing/decreasing all IKI values in the sequence, while keeping the relation between neighbouring IKI values unchanged (same shape of IKI patterns). This second scenario corresponded with timing solutions that were close-by in the movement space (Figure S3). During the last learning block, participants consistently exploited the inferred rewarded solution (Figure S4). Our reward-based motor sequence learning task was also able to capture the general effects of reward on motor exploration described in previous work^35,36^. In particular, participants explored the timing dimension more (larger unsigned changes in the IKI pattern trial to trial) following a drop in scores than after an increase in scores (Figure S5 and Supplementary Materials). A more detailed assessment of trial-to-trial reward-based motor learning used a mathematical model of the behaviour described below.

**Figure 3:**
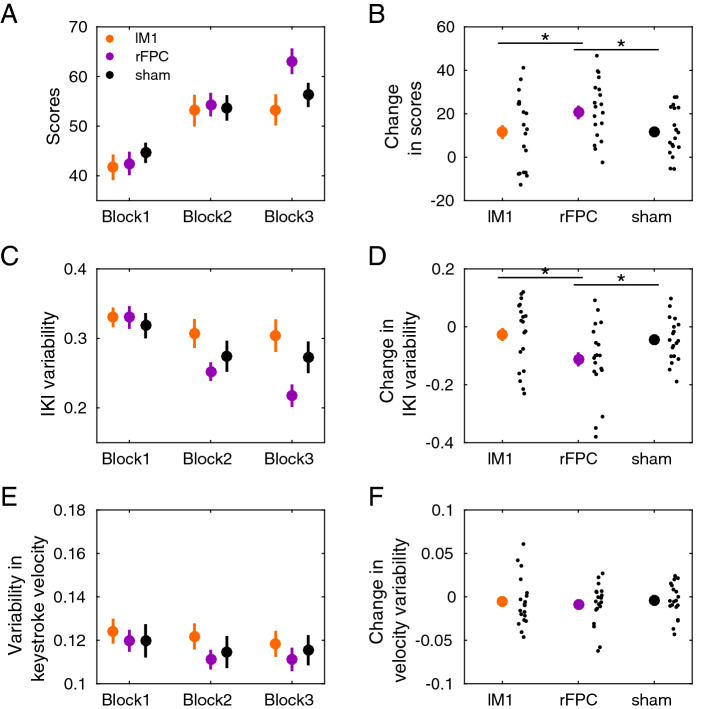
Behavioural results during reward-based learning. (**A**) Participants increased their scores across blocks (significant main effect of Block in full factorial analysis using factors Block (1–3) and Stimulation (lM1, rFPC, sham), supporting they successfully used the trial-by-trial feedback to approach the hidden performance goal. (**B**) The change in scores from online to offline blocks (3 minus 1) was significantly larger in rFPC-tDCS than sham, and in rFPC-tDCS relative to lM1-tDCS (denoted by the asterisk; $P \le P_{FDR} = 0.0414$; moderate effect sizes: $\Delta _{dep} =$ 0.67, CI = [0.61, 0.75] for rFPC-tDCS and lM1-tDCS; $\Delta _{dep} =$ 0.62, CI = [0.50, 0.80] for rFPC-tDCS and sham). (**C**) Same as (**A**) but for the degree of temporal variability (IKI variability, dimensionless). The full factorial analysis demonstrated significant main effects of Block and Stimulation. (**D**) The reduction from block 1 to 3 in IKI variability was significantly more pronounced in rFPC-tDCS than lM1-tDCS ($P \le P_{FDR} = 0.0126$, $\Delta _{dep} =$ 0.63, CI = [0.53, 0.85]), and also in rFPC-tDCS relative to sham ($P \le P_{FDR} = 0.0126$, $\Delta _{dep} =$ 0.73, CI = [0.61, 0.82]). (**E**,**F**) Same as (**C**,**D**) but for keystroke velocity. No significant main effects or interactions were found when assessing variability in keystroke velocity. Neither were there differential effects of stimulation on the change in this variable from block 1 to 3. Small black dots represent individual participant data. Coloured dots display mean values with error bars denoting ± SEM.

Statistical analysis during reward-based learning demonstrated that participants improved their scores across blocks in all tDCS conditions (Fig. 3A; main effect Block, $P = 0.0002$, full $3 \times 3$ non-parametric factorial analysis). These data support that participants successfully used the trialwise feedback in all stimulation conditions to learn about the hidden goal. There was no significant main effect for Stimulation or interaction effect ($P > 0.05$). The increase in scores across blocks observed in all tDCS sessions was not affected by potential carry-over effects. Indeed, participants did not perform the same pattern of IKI values across the separate tDCS sessions (IKI general profile: Figure S6). Week on week, participants did not exhibit a tendency to learn faster about the hidden performance target (a phenomenon called savings, see Figure S7^37^), which further supports the absence of carry-over effects in reward-based motor learning across sessions.

A planned one-way factorial analysis on the change in scores from online to offline blocks (3 minus 1) across stimulation conditions demonstrated a significant effect of factor Stimulation ($P = 0.0355$). Post hoc pair-wise comparisons for each pair of tDCS stimulation conditions revealed a significantly larger increase following rFPC-tDCS relative to lM1-tDCS (Fig. 3B; increase of 20.6 [standard error of the mean or SEM 3.72] for rFPC-tDCS; increase of 11.6 [3.8] for lM1-tDCS: $P \le P_{FDR} = 0.0414$; moderate effect size, assessed with a non-parametric effect size estimator for dependent samples^38^, $\Delta _{dep} =$ 0.67, confidence interval or CI = [0.61, 0.75]). There was also a significantly larger increase for rFPC-tDCS relative to sham ($P \le P_{FDR} = 0.0414$; moderate effect size, $\Delta _{dep} =$ 0.62, CI = [0.50, 0.80]; the average score change for sham was 11.7 [2.43]). No differences between lM1 and sham were found ($P > 0.05$).

The general increase in scores across blocks was paralleled by a reduction in the expression of task-related motor variability for timing, measured with the cv index (Fig. 3C; significant main effect of Block in the full $3 \times 3$ non-parametric factorial analysis; $P = 0.0156$). Notably, we also found a significant main effect of factor Stimulation ($P = 0.0260$), but no interaction effect. A separate one-way factorial analysis on the difference between offline and online blocks in timing variability showed a significant effect of Stimulation ($P = 0.0246$, Fig. 3D). Post hoc analyses on this difference measure revealed that following rFPC-tDCS the drop in temporal variability from online to offline learning blocks was more pronounced than following sham ($P \le P_{FDR} = 0.0126$, $\Delta _{dep} =$ 0.73, CI = [0.61, 0.82]) and also relative to lM1 ($P \le P_{FDR} = 0.0126$, $\Delta _{dep} =$ 0.63, CI = [0.53, 0.85]). When comparing lM1-tDCS to sham, however, the reduction in motor variability did not differ statistically ($P > 0.05$).

Finally, control analyses carried out on the variability in keystroke velocity, revealed no significant main or interaction effects (Fig. 3E, full $3 \times 3$ factorial analysis). Thus, variability in this non-task related dimension was not significantly modulated by stimulation or learning block (see also Fig. 3F). Additional details on general performance variables are presented in Supplementary Results.

### Modelling results

We investigated how individuals adapted their task-related behaviour as a function of the expectation of reward using a hierarchical Bayesian model, the Hierarchical Gaussian Filter for continuous inputs (HGF^15,39^). The HGF was adapted to model participants’ beliefs about the reward on the current trial *k*, $x^k_1$, and about its rate of change, termed environmental volatility, $x^k_2$. In the HGF for continuous inputs, the true volatility state varies as a function of the changes in the true value of the reward tendency. However, participants’ *beliefs* on volatility could also be modulated by the uncertainty associated with inferring the performance-to-score mappings (Figure S3; section “Reward function”), as timing patterns close-by in the movement space lead to different rewards. Volatility was not experimentally manipulated as in previous decision-making studies, where the reward mapping changed every block^15,40,41^.

In the HGF, the update equations for the mean of the posterior distribution of beliefs on reward ($\mu _1$) and log-volatility ($\mu _2$) depend on the corresponding prediction errors (PE) weighted by precision (pwPE; precision being the inverse variance or uncertainty of the posterior distribution, “Materials and methods”). The perceptual HGF model was complemented with a response model, which defines the mapping from the trajectories of perceptual beliefs onto the observed responses in each participant. We were interested in assessing how belief trajectories or related computational quantities (e.g. pwPEs) influenced subsequent behavioural changes, such as trial-to-trial timing variability or average tempo. The response models, accordingly, explained in each participant a behavioural dependent variable (*Y*) with two predictor computational variables ($X_1, X_2$), modulated by the regression coefficients ($\beta _1$, $\beta _2$, and the intercept $\beta _0$): $Y = \beta _0 + \beta _1X_1 + \beta _2X_2 +\zeta$, with $\zeta$ representing the residual term.

**Figure 4:**
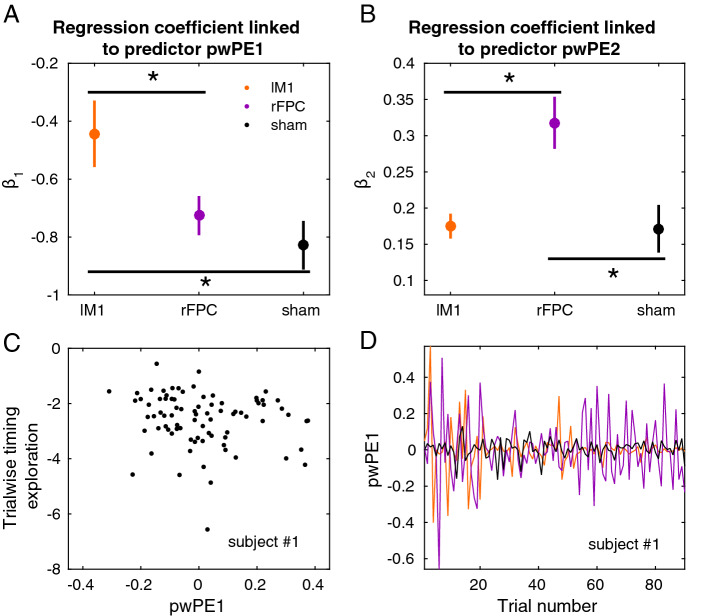
Computational modelling analysis. Data shown as mean and ± SEM. (**A**,**B**). $\beta$ coefficients of the response model that explains the behavioural changes in trial *k* as a linear function of the precision-weighted prediction errors (pwPE) updating beliefs on reward (pwPE1) and volatility (pwPE2) on the previous trial, $k-1$. The performance measure assessed trialwise timing exploration. It was computed as log($|\Delta$cvIKI|), which is the unsigned *change* from trial $k-1$ to *k* in the degree of timing variation across keystroke positions. See “Materials and methods”. (**A**) There was a significant main effect of factor Stimulation on $\beta _1$ (one-way non-parametric factorial analysis, $P = 0.004$). (**B**) Coefficient $\beta _2$ was also modulated significantly with the factor Stimulation (one-way non-parametric factorial analysis, $P = 0.012$). Differences between pairs of stimulation conditions in $\beta$ coefficients, as found in post-hoc analyses, are denoted by the horizontal black line and the asterisk ($P \le P_{FDR}$, see main text). (**C**) Illustration of the association between trialwise pwPE on reward and the subsequent task-related exploration, log($|\Delta$cvIKI|), in one subject during sham. (**D**) Illustration of the trajectories of pwPE on reward across all stimulation conditions in the same subject.

Among different alternative response models, random effects Bayesian model selection provided stronger evidence for the response model that explained changes in timing variation in the *current* trial as a linear function of pwPEs updating estimates on reward and log-volatility on the *preceeding* trial (see Fig. 4 and “Materials and methods”). Simulations of this model revealed that agents observing a broader range of scores or introducing higher trial-to-trial exploration (larger absolute changes in the trialwise timing pattern) have greater expectation on volatility, as both contributed towards an increased rate of change in the expectation on reward (Figure S9). On the other hand, in agents who steadily increased the achieved scores there was a drop in the log-volatility estimate, as the environment (and changes in the reward tendency) became more stable.

In the winning model, all $\beta$ coefficients of the multiple linear regression response model for the trialwise timing exploration were significantly different from zero in each stimulation condition ($P \le P_{FDR} = 0.001$; Fig. 4A,B). On average $\beta _1$ was negative. This outcome indicated that larger pwPEs updating reward estimates on the previous trial (pwPE1; increasing the expectation of reward) promoted an attenuation in motor exploration (reduced trial-to-trial unsigned changes in timing). That is, increases in the expectation of reward were followed by exploitative behaviour in the relevant variable (Fig. 4C,D), as expected^35,36^. A non-parametric one-way factorial analysis demonstrated a significant effect of Stimulation on the $\beta _1$ coefficients ($P = 0.004$). Post hoc analyses further revealed that lM1-tDCS decreased the sensitivity of this association relative to sham and also when compared to rFPC-tDCS ( reduced “negative” slope, larger $\beta _1$ values in lM1-tDCS than sham, $P \le P_{FDR} = 0.003$, $\Delta _{dep} = 0.68$, CI = [0.57, 0.83]; similar outcome for the lM1-tDCS and rFPC-tDCS comparison: $P \le P_{FDR} = 0.003$, $\Delta _{dep} = 0.62$, CI = [0.55, 0.80]; Fig. 4A).

The effect of pwPEs updating log-volatility (pwPE2) on trial-to-trial task-related exploration was also dissociated between stimulation conditions (significant effect of Stimulation in one-way factorial analysis, $P = 0.012$; Fig. 4B). Post hoc analyses additionally demonstrated a dissociation between rFPC-tDCS and sham stimulation in this parameter, as $\beta _2$ coefficients were positive and significantly larger for rFPC relative to sham stimulation ($P \le P_{FDR} = 0.003$; $\Delta _{dep} = 0.68$, CI = [0.55, 0.83]). Accordingly, a larger pwPE updating log-volatility on the previous trial—related to an increase in the expectation of volatility—was followed by more pronounced exploration for rFPC-tDCS than sham. Conversely, negative pwPE2 promoted greater exploitation in rFPC-tDCS. A similar dissociation was found when comparing both active stimulation conditions, rFPC-tDCS and lM1-tDCS, due to significantly larger $\beta _2$ coefficients in rFPC-tDCS than in lM1-tDCS (positive coefficients; $P \le P_{FDR} = 0.003$; $\Delta _{dep} = 0.71$, CI = [0.58, 0.89]). These results indicate that for rFPC-tDCS the sensitivity (slope) of this association was greater than for sham or lM1. Accordingly, rFPC-tDCS was associated with a more pronounced tendency to exploit the inferred timing solution (smaller trialwise timing exploration) with decreased volatility estimates. Thus, the winning response model identified different behavioural strategies in response to updates in reward and volatility as a function of the tDCS stimulation condition.

### Electric field distribution of tDCS

**Figure 5:**
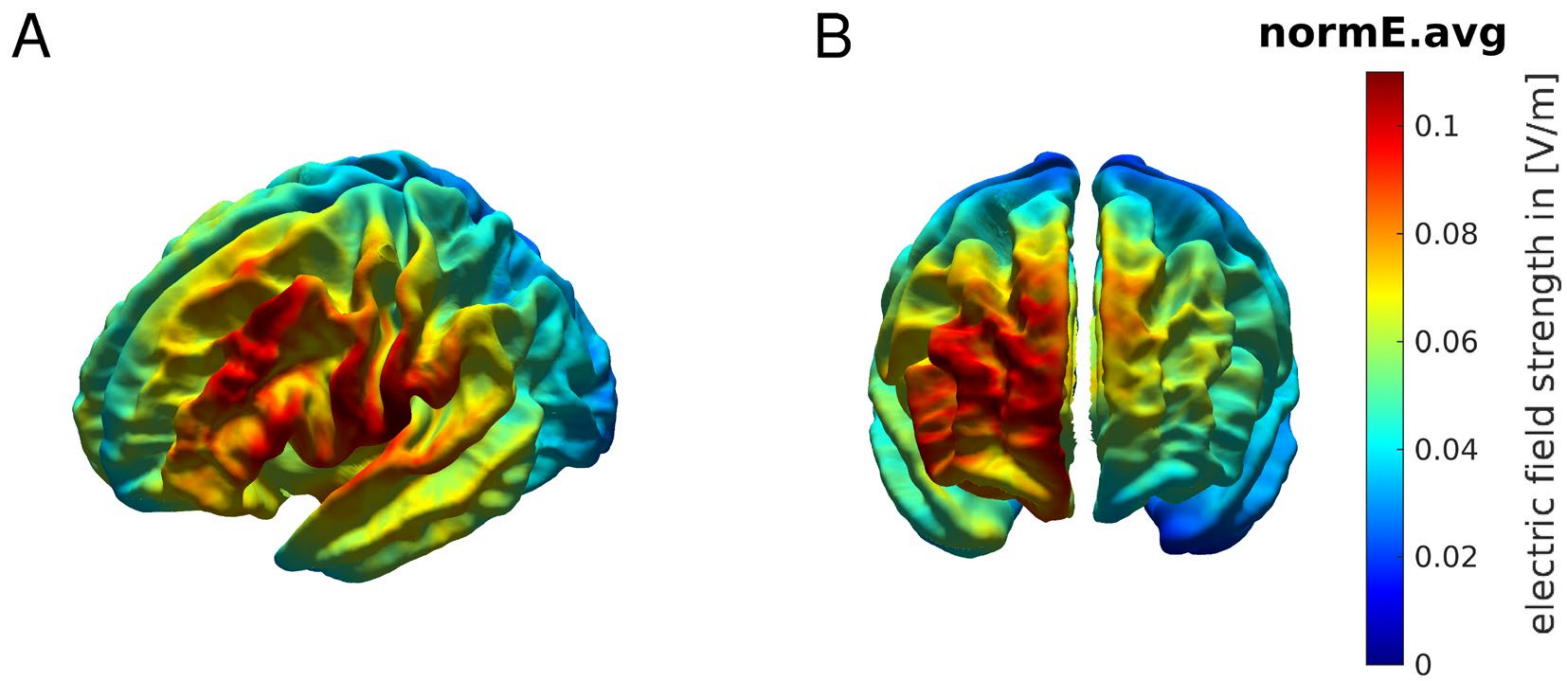
Electric field distribution for anodal lM1-tDCS (**A**) and rFPC-tDCS (**B**). Norm of the electric field strength (normE) derived from FEM calculations using SimNIBS, and averaged across participants using the fsaverage-transformed surface. Figure created with the freely available SimNIBS 2.1 software^30,31^, https://simnibs.github.io/simnibs.

To control for the confound that anodal tDCS likely increases cortical excitability but could also lead to the opposite polarity of the effect^42^, we complemented the main analysis with a simulation of the electric field induced by tDCS in each participant using SimNIBS (^30,31^; see “Materials and methods”: tDCS). This analysis focused on the focality and magnitude of the neuromodulatory effects induced by the active tDCS protocols. The results revealed that the focus of the induced electric field was within the targeted regions and had a similar magnitude in both structures (Fig. 5). The peak values of the vector norm of the electric field (normE) did not differ between active stimulation conditions ($99.9\%$ percentile: mean and SEM for lM1-tDCS = 0.132 [0.006] V/m; for rFPC-tDCS = 0.134 [0.010] V/m; permutation test, $P > 0.05$). In addition, the volume corresponding with the $99.9\%$ percentile of the field strength was not significantly different between active tDCS conditions (focality: 1.22 [0.11] $\times 10^4\, {\mathrm {mm}}^3$ for lM1-tDCS; 1.14 [0.08] $\times 10^4\, {\mathrm {mm}}^3$ for rFPC-tDCS; $P > 0.05$). Notwithstanding the similarity in peak and focality of the simulated normE values for lM1 and rFPC-tDCS, in both cases the electric field spread to neighboring areas beyond the target coordinate. Under lM1-tDCS, the induced electric field was maximum in the left M1 (area 4 of the human connectome project multi-modal parcellation, HCP-MMP1^43^), followed by the premotor cortex (6), prefrontal areas (8Av and 8C) and somatosensory cortex (3). Under rFPC-tDCS, the peak of the electric field corresponded with the rFPC (areas 10p and 10pp), followed by regions in the medial prefrontal cortex (mPFC; 9) and orbitofrontal cortex (OFC; 11). Lastly, the variability in the electric field strength (standard deviation) did not differ between tDCS targets ($P > 0.05$, Figure S11), supporting the comparable effects of both stimulation protocols in our sample.

## Discussion

In this study, we identify a potential role of rFPC in reward-based motor learning by using a motor task that requires a shift from exploration to exploitation, a Bayesian computational model of the behaviour^15^, and simulations of the electric field induced by tDCS. The results indicated that rFPC-tDCS relative to sham and lM1-tDCS accelerated the increment in scores from online to offline learning blocks primarily through regulation of task-related motor variability. Trial-to-trial analyses using computational modelling further demonstrated that across all stimulation protocols increased expectation of reward led to subsequent exploitation, as expected^4,36^. Because the sensitivity (slope) of this association was greater for rFPC-tDCS and sham relative to lM1-tDCS, these results suggest that behavioural changes following pwPEs updating reward estimates were enhanced for rFPC-tDCS and sham. Frontopolar stimulation was however dissociated from lM1 and sham stimulation with regards to the effects of trial-to-trial volatility updates on exploration. While lM1-tDCS and sham were less sensitive to updates in the expectation on volatility, rFPC-tDCS promoted greater exploitation of the inferred timing pattern as the expectation on log-volatility progressively decreased—with increasing expectation on reward. These findings suggest that rFPC contributes to reward-based motor learning by promoting a shift from exploration towards successful exploitation. This shift is mediated by an enhanced sensitivity to environmental volatility, which is associated with changes in the reward structure over time. Our results extend findings in the area of decision-making^10–13^ to that of motor skill learning: Brain regions previously linked to decision-making in the cognitive or perceptual domain, such as the FPC, could be relevant in the motor domain. The findings also complement recent tDCS work associating the dorsolateral PFC to motor decision-making^44^.

Neuromodulation of the rFPC via anodal tDCS reduced motor variability during learning blocks. This effect was specific to the learning phase, as rFPC-tDCS did not modulate baseline motor noise, similarly to lM1-tDCS and sham. Recent work has demonstrated that the inhibition of the rFPC via TMS during binary choices decreased directed exploration driven by information seeking^10^. In line with that result, cathodal versus anodal rFPC-tDCS have been shown to decrease or increase exploration, respectively, in a three-armed bandit task^11^. Thus, a surprising outcome was that, compared to lM1-tDCS and sham, anodal rFPC-tDCS did not increase motor variability during learning but instead reduced it towards the third block. This apparent discrepancy can be better understood by comparing our motor task with the decision-making tasks used in FPC studies. Binary or multi-armed bandit tasks as used in decision-making studies generally use a probabilistic reward function that changes over time^10,11,15,40^. In this context, an optimal policy should continuously balance the exploration–exploitation tradeoff^45^, while FPC might drive exploration as needed for the task demands^10,11^. On the other hand, motor tasks that use continuous reward signals to guide learning typically maintain the same reward structure over time^4,5,34^. Here, movement variability is initially high but progressively decreases as participants approach the hidden solution. In this scenario, our study demonstrated that rFPC-tDCS facilitates the decline in motor variability and an increase in scores. This is consistent with the evidence from human and non-human primates, supporting that FPC might have evolved to monitor multiple competing choices and direct the exploratory tendency towards the most rewarding one^12^. This function of FPC thus makes it particularly suitable to track the reward associated with (multiple) continuous movement parameters, such as timing or force in our study.

To understand why FPC stimulation led to the largest increase in scores from online to offline blocks, the computational results should be considered. Across blocks, there was an attenuation of the expectation on log-volatility in all stimulation conditions, which reflects that the reward tendency estimate became more stable over time. This result also indicates that over time, trial-to-trial changes in scores (and expectation on reward) were associated with increasingly more negative update steps (pwPE2) on volatility. Because under rFPC-tDCS the positive slope of the association between exploration and pwPE2 was greater than for lM1-tDCS and sham—indicating higher sensitivity in this association (larger $\beta _2$)—smaller pwPE2 values over time would lead to more exploitative behaviour in rFPC-tDCS. This finding thus dissociates the effects of lM1-tDCS and sham on reward-dependent motor learning from those of rFPC-tDCS. It hints at an important condition for successful reward-based motor learning: increased sensitivity of task-related exploration to changes in volatility.

How was the mapping between movement and reward acquired in the different stimulation protocols? In the continuous movement space over which our task was defined, timing was the task-related dimension that needed to be mapped to reward. Thus, one possibility is that rFPC-tDCS might have facilitated the acquisition of this complex mapping, increasing the achieved scores and the expectation on reward, and reducing volatility estimates. This remains speculative at this moment as a limitation of this study is that we did not track neural dynamics during task performance. Therefore, we could not assess whether rFPC-tDCS modulated the emergence of a neural representation of the mapping between movement parameters and reward. On the behavioural level, however, the results converge in showing that rFPC-tDCS led in parallel to increased task-related exploitation and increased scores. Accordingly, the timing pattern that was exploited under frontopolar stimulation was indeed closer to the hidden target than the solutions inferred under sham and lM1-tDCS. The results are consistent with previous findings indicating that FPC infers the absolute reliability of several alternative goals^46,47^. Moreover, FPC manages competing goals by keeping track of alternative choices^12,13^. Notably, our results expand previous findings by revealing that during motor learning, which is generally solved in a continuous movement space^7^, learning the mapping between different movement configurations and their associated reward partially relies on FPC, although an additional involvement of the dorsolateral PFC is also likely^44^.

Stimulation over the contralateral M1 did not modulate motor variability across blocks (IKI variability) when compared to sham stimulation. This lack of significant effects should be interpreted with caution as our statistical approach does not allow us to make inferences on null results. However, in the modelling analysis, motor cortex stimulation was associated with a reduced sensitivity to changes in the expectation of reward. Although we had hypothesised that rFPC would accelerate reward-based learning more when compared to lM1-tDCS, which we confirmed, we also predicted that lM1-tDCS would increase exploration and improve learning from reward signals relative to sham. This prediction was based on the existing TMS and tDCS studies demonstrating the involvement of M1 in reward-based motor learning^27,48^ and decision-making^25,26^. Furthermore, M1 contributes to reward-based motor learning via neurophysiological plasticity changes, such as long-term potentiation^27^. This would be consistent with the maximum electric field intensity over lM1 in our study indicating excitatory effects, which relate to a decrease in local GABAergic activity and enhanced long-term potentiation-like activity^17,49,50^. It is noteworthy that most previous studies linking M1 to reward-based motor learning (see above), reward-guided motor processing^20,21^ and valued-based decision making^22,23^ used TMS protocols. Accordingly, the focality of TMS may be necessary to overcome the large inter-individual variability affecting tDCS studies^42^. Arguing similarly^24^ used smaller electrodes anterior and posterior to M1 to demonstrate a benefit of reward signals and M1-tDCS on motor retention. Thus, resolving the issue of the dissociable role of M1 and FPC in regulating motor variability during reward-based motor learning will require follow-up TMS studies and, additionally, a comparison between initial learning and motor retention^51^. Because the largest effects of rFPC relative to lM1-tDCS emerged when contrasting the achieved scores in offline versus online blocks, future work should clarify whether the benefits of rFPC over lM1-tDCS during reward-based motor learning are specific to offline stimulation, as M1-tDCS effects on motor learning may be limited to online stimulation^18,29^. Lastly, follow-up studies should also address whether the differential effects of rFPC and lM1 stimulation on the regulation of motor variability can be accounted for by a potentially delayed role of M1 in the refinement of task-related behavioural variability^19^.

Of note, a limitation of any tDCS study is that the diffuse spatial effect of tDCS does not allow us to determine whether modulation of the target area alone is responsible for the observed behavioural effect^42^. Even with the higher spatial resolution of TMS, it has been argued that any effect of FPC stimulation on behaviour could be mediated by other brain regions coupled with FPC during behavioural exploration^10^, such as the inferior parietal cortex or ventral premotor cortex^46^ or by regions engaged in other aspects of goal-directed behaviour, such as the ventromedial or dorsolateral PFC and OFC^12,16,44^.

To mitigate that limitation, we modelled the electric field in the individual anatomy and assessed the strength and focality of the induced electric field for each tDCS target. We found an enhanced focal activation with maxima in the targeted areas for both active stimulation protocols. Recent work demonstrated an association between enhanced electric field strength in SimNIBS due to anodal tDCS and excitatory effects^49^, however their results are limited to M1. Accordingly, neurophysiological implications for rFPC stimulation cannot be drawn out at this point. Future studies combining electroencephalography and functional MRI should assess the network of interactions between FPC, other regions in the PFC, and cortical motor regions to determine the precise mechanism underlying the rFPC-tDCS effect on reward-based motor learning reported here.

## Materials and methods

### Participants

Nineteen right-handed participants (10 females, mean = 27.7 yrs, std = 3.3 years, range 21–33) with no history of neurological disease or hearing impairment and with no musical training outside of the requirements of the general music curriculum in school were recruited. Laterality quotient was assessed by the Oldfield handedness inventory (^52^; mean = 90, standard error of the mean or SEM = 3.2; values available in 17/19 participants). The sample size is small but similar to that found in tDCS studies focusing on motor learning^24,53^ and was based on our previous estimation of the minimum sample size required to detect effects of different experimental manipulations in this paradigm (e.g. reward or affective manipulations^14^) with a statistical power of 0.95. The study protocols were approved by the local ethics committee of the of the University of Leipzig (277-14-25082014) and agrees with the provisions of the Helsinki Declaration^54^. Participants gave written informed consent before the beginning of the first experimental session. To incentivise participants during completion of the reward-based learning phase of the motor task, they were informed about a €50 voucher for online purchases that would be awarded to the participant scoring the highest average score across the three sessions.

### Experimental design and procedure

A sham-controlled, double-blinded, cross-over design was implemented. The study was comprised of three sessions with a 7-days interval between them (same time of day) to reduce potential carry-over effects. Selection of target coordinates for each tDCS protocol was guided by individual T1-MRI. To improve the blinding procedure and prevent any systematic influences of sham effects on behaviour, rFPD-tDCS or lM1-tDCS montages were pseudo-randomly used as sham-tDCS montages, counterbalanced across participants.

In the learning phase, participants were explicitly instructed to vary the timing of the performance as this dimension was associated with reward. The instructions they received were (approximate translation from German): “The winning solution associated with 100 points is a specific combination of short and long time intervals between consecutive key presses. Try different combinations of short and long intervals until you discover the solution that gives you the most points”. We implemented a mapping rule between movement and reward governed by uncertainty (see next section), as in our task different timing patterns could receive the same reward, whereas similar timing patterns would obtain different rewards (Figure S3). This choice was based on previous research suggesting that the acquisition of complex motor skills in daily life often involves an uncertain or variable mapping between actions and outcomes^55^. Moreover, higher reward uncertainty can be beneficial for motor retention^55^. In our task, the optimal strategy to maximise the mean total reward involves two phases: (i) an initial increase in exploration to learn the mapping between reward and movement parameters, followed by (ii) a swift switch to the exploitation of the performance inferred as most rewarding. The quantitative analysis of exploration is described below. Prior to receiving tDCS and completing the motor task, participants had to familiarise themselves with the series of tones they had to produce during each phase of the task. We introduced auditory feedback to make the task more engaging and to facilitate the memorisation of the sequence content (i.e. to reduce production errors). This choice was based on previous research showing that non-musicians learn audio-motor sequences better than visuo-motor (i.e. silent) sequences of finger presses^56^. The stimulus material in the baseline phase consisted of a series of eight consecutive white piano keys played with four fingers (four notes upwards + same four notes downwards; one finger per key with fixed finger-to-key mapping). In the learning phase, the stimulus material was comprised of a sequence of eight notes, which was a combination of the four neighboring white keys they had to press during the baseline phase (Figure S1). Three different types of sequences were used for each stimulation session, with a pseudo-randomised counterbalanced order across participants. Each sequence was defined over a similar range of semitones but the range had a different spatial location on the keyboard (i.e. towards higher or lower pitch values, Figure S1). Participants were explicitly taught the order of the notes for the baseline and reward-based learning phase by one of the experimenters, who played the notes for the participants using an isochronous timing. Participants had to repeat the sequence of notes after the demonstration by the experimenter using a self-paced tempo. Because the stimulus materials for both phases were short and the order of the notes easy to play, all participants demonstrated an error-free performance after just a few repetitions (baseline materials: 2 repetitions on average, range 1–4; learning materials: 4 repetitions on average, range 2–6).

Once under tDCS, the baseline phase required participants to press the corresponding series of white keys regularly at a self-paced tempo. This phase allowed us to assess fine motor control during regular performance as a proxy for baseline motor noise.

Next, during the reward-based learning blocks, participants had to play the corresponding sequence of notes (Figure S1). Crucially, however, at this point they were instructed that the timing of the performance target was not isochronous and thus their goal was to use trial-based feedback scores to discover and approach that hidden target. Participants were not aware that different timing solutions could receive the same reward.

### Reward function

The performance measure that was rewarded during learning blocks was the Euclidean norm of the vector corresponding to the pattern of temporal differences between adjacent inter-keystroke-intervals (IKI, in s) for a trial-specific performance (as in^14^). To approach the hidden target performance, participants had to deviate from an isochronous performance and find out the right combination of successive IKIs.

Here we denote the vector norm by $\Vert {\varvec{\Delta }}{\mathbf {z}}\Vert$, with ${\varvec{\Delta }}{\mathbf {z}}$ being the vector of differences, ${\varvec{\Delta }}{\mathbf {z}} = ( {{\mathrm{z}}_2 - {\mathrm{z}}_1, {\mathrm{z}}_3 - {\mathrm{z}}_2,\ldots , {\mathrm{z}}_n - {\mathrm{z}}_{n-1}})$, and ${\mathrm {z}}_{\mathrm{i}}$ representing the IKI at each keystroke (i = 1, 2,..., n). Notably, IKI values represent the difference between the onset of consecutive keystrokes, and therefore ${\varvec{\Delta }}{\mathbf {z}}$ indicates a vector of differences of differences (put simply: differences of intervals). The target value of the performance measure for each sequence was a vector norm of 1.9596 (e.g. one of the maximally rewarded performances leading to this vector norm of IKI-differences would consist of IKI values: [0.2, 1, 0.2, 1, 0,2, 1, 0.2] s; that is a combination of short and long intervals). The score was computed in each trial using a measure of proximity between the target vector norm $\Vert {\varvec{\Delta }}{\mathbf {z}}^t\Vert$ and the norm of the performed pattern of IKI differences $\Vert {\varvec{\Delta }}{\mathbf {z}}^p\Vert$, using the following expression:

1 $$\begin{aligned} {\mathrm {score}} =100 \,{\mathrm {exp}}( - \left|\Vert {\varvec{\Delta }}{\mathbf {z}}^{t}\Vert -\Vert {\varvec{\Delta }}{\mathbf {z}}^{p}\Vert \right|). \end{aligned}$$

As mentioned above, different combinations of IKIs could lead to the same IKI differences and thus same Euclidean norm. Thus, timing patterns that were far away in the movement space could receive the same reward, whereas timing patterns close-by in the movement space would obtain different rewards (Figure S3). Accordingly, the mapping rule between movement and reward was governed by uncertainty, and higher overall exploration could be associated with the perception that the environment and reward structure was more unstable. This was explicitly assessed in the mathematical model of the behaviour described below.

### tDCS

During the experiment, tDCS was applied via saline-soaked sponge electrodes to the individual target coordinate using a battery-driven DC-stimulator (NeurConn, Ilmenau, Germany). tDCS can transiently modulate cortical excitability via application of direct currents, as shown in combined TMS-tDCS studies^17,57^, and further supported by recent simulation studies^49^. Anodal tDCS has been shown to increase cortical excitability, however additional evidence indicates that it could lead to the opposite polarity of the effect, reducing cortical excitability^42^. For instance, extending the stimulation duration beyond 26 min has been shown to result in inhibitory rather than excitatory effects after anodal tDCS^58^. To control for this confound, we complemented the main analysis with a simulation of the electric field induced by tDCS in each participant using SimNIBS (see below). Anodal or sham tDCS was applied to the right FPC or left (contralateral) M1 regions. The target coordinate for rFPC-tDCS was selected from previous tDCS and fMRI work investigating the role of rFPC on exploration (Montreal Neurological Institute or MNI peak: x = 27, y = 57, z = 6^11,16^). For lM1-tDCS, we used a target coordinate in the hand area of the left primary motor cortex (MNI peak: x = − 37, y = − 21, z = 58), based on^59^. The target coordinates were transformed to the individual native space using a T1-weighted high-resolution magnetic resonance image from each participant. Specifically, the MNI coordinates were converted into participants’s native MNI space using the reverse native-to-MNI transformation from Statistical Parametric Mapping (SMP, version SPM12). The point on the scalp corresponding with each of our targeted brain areas was marked to place the active electrode (5 $\times$ 5 cm$^2$). The reference (cathode, 10 $\times$ 10 cm$^2$) electrode for rFPC-tDCS was placed at the vertex^11^, whereas it was located over the frontal orbit for lM1-tDCS^17^. Flexible elastic straps were used to fixate the electrodes on the head. A three-dimensional (3D) neuronavigation device (Brainsight Version 2; Rogue Research, Montreal, Canada) was used to guide positioning of active electrodes.

We stimulated with a weak direct current of 1 mA in all conditions for 20 min resulting in a current density of 0.04 mA/cm^2^ under the target electrode and 0.01 mA/cm^2^ under the reference electrode. In general, the modulatory effect of tDCS on brain excitability is more pronounced after several minutes and can subsequently outlast the stimulation duration for up to 1.5 h^17,57^. To account for the potential delay in the effect of tDCS, we instructed participants to wait for 3 min before we initiated the motor task. At the start of the active tDCS stimulation the current was ramped up for 30 s to minimise the tingling sensation on the scalp, which generally fades over seconds^18^, and was also ramped down for 30 s at the end. During sham tDCS, the current was ramped-up for 30 s, held constant at 1 mA for 30 s and ramped-down for 30 s. This procedure aimed to induce a similar initial tingling sensation in active and sham protocols, yet without modulation of cortical excitability for sham tDCS^60^. Details on the double-blind procedure are presented in the Supplementary Materials.

Before and after each tDCS session, participants rated on a 1–10 scale their fatigue, attention and discomfort levels, as well as the sensation with tDCS (post-tDCS, scale 1–5). No significant differences between active and sham tDCS sessions were found in the reported levels of fatigue, discomfort or attention levels ($P > 0.05$, post minus pre changes; paired permutation test). The sensation was not different between rFPC and sham tDCS, either ($P > 0.05$). However, lM1-tDCS induced a higher sensation than sham (P = 0.0034, non-parametric effect size $\Delta _{dep} =$ 0.87, CI = [0.57, 0.88]. Details on statistical methods are provided below).

### Acquisition and analysis of behavioural data

Performance information was saved as MIDI (Musical Instrument Digital Interface) data, which provided the time onsets of keystrokes relative to the previous event (inter-keystroke interval, IKI, s), MIDI note number that corresponds with the pitch, and MIDI velocity (related to loudness). Behavioural data are available in the Open Science Framework Data Repository: https://osf.io/zuab8/.

The assessment of motor variability along each dimension (time, keystroke velocity) was performed by computing the coefficient of variation (cv = std/mean, across trials within the block) for each variable at each keystroke position, and then averaging the values across keystroke positions. During the baseline phase participants had to accurately reproduce the same action (regular timing and keystroke velocity). In this context, any residual variability can be regarded to reflect motor noise^4,32^, which here was measured assessing variability in IKI and keystroke velocity in this initial phase. During learning blocks, the level of task-related motor variability, IKI variability, was considered to primarily reflect intentional exploration of this parameter but also some degree of unintentional motor noise—similarly to other studies^4,5,14^. Participants were instructed that the keystroke velocity of their performance was not related to reward. Thus, changes in variability of keystroke velocity across blocks—if present—would be an indication of changes in unintentional motor noise with learning. The achieved scores and other general performance variables, such as the block-wise mean tempo, mean keystroke velocity and rate of wrong notes (error rate) were also evaluated.

During the baseline phase we assessed statistically the effects of stimulation conditions on the relevant behavioural variables (see “Results”), excluding the scores. During the learning blocks, statistical analysis focused on the investigation of the effect of stimulation and learning block on all behavioural dependent variables. In addition, we were specifically interested in assessing the influence of tDCS protocols on the change in task-related motor variability and scores from online to offline (after the cessation of tDCS) learning blocks. Thus, additional dependent variables were the difference between blocks 3 and 1 in IKI variability and, separately, the scores. Details on statistical testing are provided in section “Statistical analysis”. When providing mean values on behavioural variables, we also indicate the standard error of the mean or SEM.

### Bayesian model of behaviour

**Figure 6:**
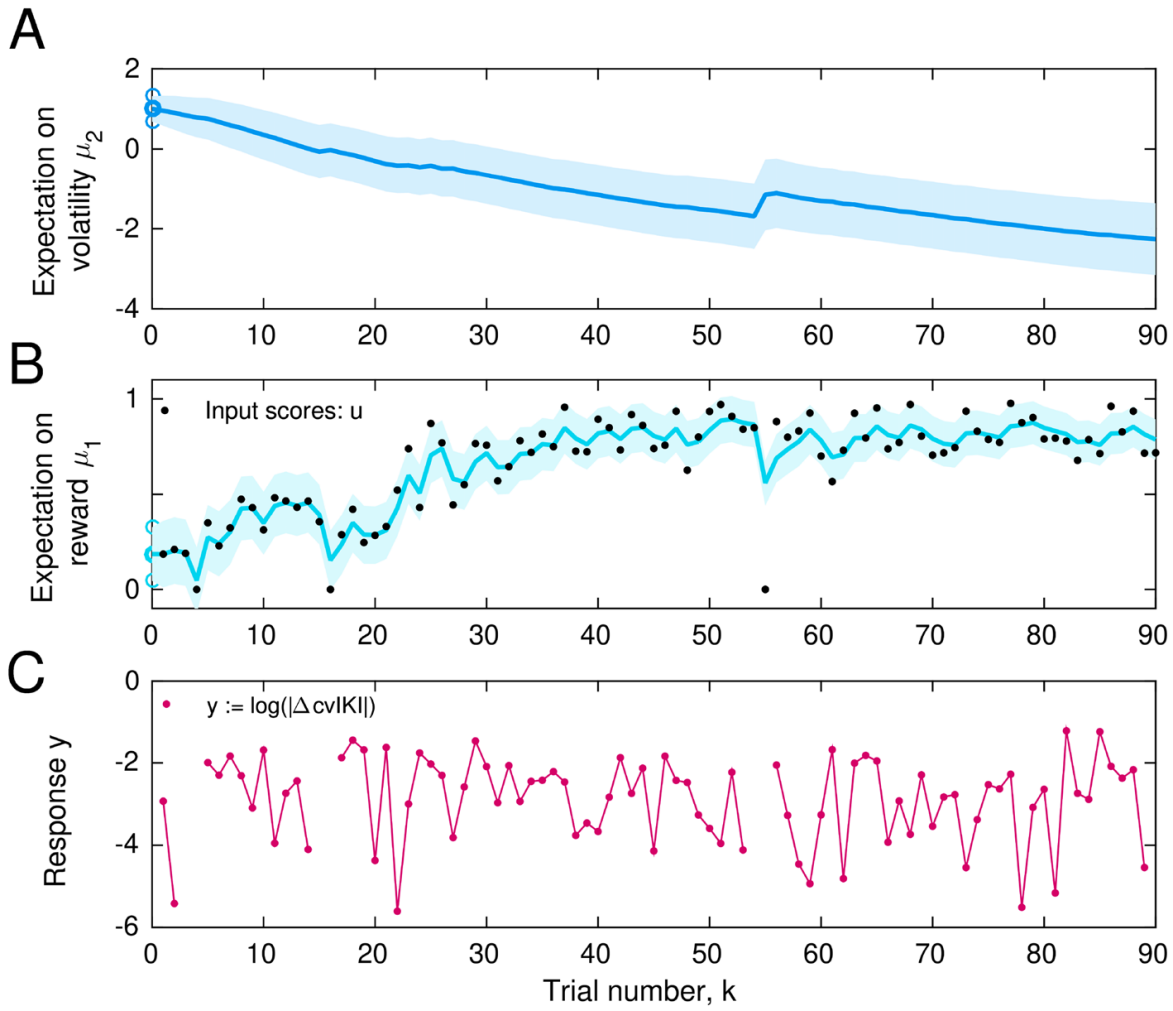
Computational model: output trajectories. (**A**) Example of trial-by-trial beliefs about volatility, with posterior mean $\mu _2$ (variance $\sigma _2$) in the perceptual HGF. (**B**) Belief on the first level, which represents an individual’s expectation of reward, $\mu _1$ (variance $\sigma _1$). Black dots represent the trialwise input scores (*u*). (**C**) Performance output (logarithm of the unsigned change in trialwise ${\mathrm {cvIKI}}$ as a proxy for exploration). Shaded areas denote the variance or estimation uncertainty on that level.

In the HGF model for continuous inputs we implemented^15,39^, beliefs on $x_1$ and $x_2$ were Gaussian distributions and thus fully determined by the sufficient statistics $\mu _i$ ($i = 1, 2$, mean of the posterior distribution for $x_i$, corresponding to participants’ expectation) and $\sigma _i$ (variance of the distribution, representing uncertainty of the estimate). The belief trajectories about the external states $x_1$ and $x_2$ (mean, variance) were further used to estimate the most likely response corresponding with those beliefs. An illustration of the model output trajectories is shown in Fig. 6. See also Figure S8 for a schematic of the modelling approach with the relevant variables and parameters.

The update equations for the posterior mean of the belief distribution at level *i* and for trial *k*, $\mu _{i}^{k}$, are included in the Supplementary Materials online. Detailed definitions can also be found in^14,15,39^. Details on the free paratemers of the HGF model to be estimated in each individual are presented in the Supplementary Materials online. In addition, Table S1 shows our choice of prior values on the HGF parameters that were used to generate belief trajectories.

The response model defines the mapping from the trajectories of perceptual beliefs onto the observed responses in each participant. We were interested in assessing how belief trajectories or related computational quantities influenced subsequent behavioural changes, such as trial-to-trial variability or exploration. Accordingly, we constructed different response models associated with different scenarios in which participants would link a specific performance measure to reward, such as the mean duration of key presses or the degree of timing variation across keystroke positions (measured with the trialwise cvIKI across presses). Response variables that were bounded to 0–1 in their native space, such as $|\Delta$ cvIKI$^{k}|$, were transformed into an unbounded variable using the logarithmic transformation. See details on the Supplementary Materials online.

For each performance measure, the corresponding response model explained that variable as a function of (a) the mean of the posterior distribution of beliefs $\mu _1$, or $\mu _2$; (b) the precision-weighted PE (pwPE) about reward, pwPE1, or volatility, pwPE2; (c) HGF quantities related to beliefs on the reward tendency: $\mu _1$, pwPE1; (d) HGF quantities related to volatility estimates: $\mu _2$, pwPE2. This led to a total of 16 different models. The rationale for choosing pwPE1 and pwPE2 as predictors in some of the alternative response models was the relevance of PE weighted by uncertainty in current frameworks of Bayesian inference^61,62^. Moreover, pwPEs determine the step size of the update in the expectation of beliefs (see Supplementary Materials online). That is, larger pwPEs about reward increase the expectation of reward, while larger pwPE about volatility increase the corresponding volatility estimate. See^14^ for a similar use of the response models defined for this paradigm.

Each model was fitted with the 90 trialwise performance values of the corresponding response variable and with the input scores for each tDCS session. The log model-evidence (LME) was used to optimise the model fit^63^. Random Effects Bayesian Model Selection (BMS; code freely available from the MACS toolbox^64^) was performed across all 16 models using the LME values. BMS provided stronger evidence for the response model that explained log($|\Delta$ cvIKI$^{k}|$) as a linear function of pwPE about reward, pwPE1 (termed $\epsilon _1$ in Eq. (2)), and volatility, pwPE2 (termed $\epsilon _2$), on the preceeding trial $k-1$:

2 $$\begin{aligned} {\mathrm {log}}(|\Delta {\mathrm {cvIKI}}^{k}|) = \beta _0 + \beta _1 \epsilon _{1}^{k-1} + \beta _2 \epsilon _{2}^{k-1} + \zeta , \end{aligned}$$

where $\zeta$ is a Gaussian noise variable. The response variable log($|\Delta$ cvIKI$^{k}|$) reflected unsigned changes (exploration) from trial $k-1$ to trial *k* in cvIKI^35^. In this winning model, the exceedance probability was 0.9888 and the model frequency was $72\%$.

The HGF with the winning response model provided a good fit to the behavioural data, as the examination of the residuals shows (Figure S10). There were no systematic differences in the model fits across tDCS conditions. The response model noise parameter $\zeta$ was not significantly modulated by the stimulation condition ($P > 0.05$; average value $\zeta$ = 1.3 [0.08]).

The effect of stimulation on the learning process, as described by the computational model, was assessed by analysing the following dependent variables: The $\beta$ coefficients of the winning response model ($\beta _1$, $\beta _2$) that regulate how pwPE on reward and volatility modulate task-related behavioural adaptations, as well as the noise parameter $\zeta$. This analysis thus allowed us to investigate how trial-to-trial update steps in the expectation of reward and volatility (related to changes in observed scores) modulated task-related motor exploration in the next trial.

### SimNIBS

The electric field distribution induced by each tDCS condition was simulated in each participant with the freely available SimNIBS 2.1 software^30,31^. SimNIBS integrates different tools, such as FreeSurfer, FMRIB’s FSL, MeshFix, and Gmsh^65^. Using the headreco head modelling pipeline of SimNIBS, the electrically most relevant tissue structures (skin, skull, cerebrospinal fluid, gray matter, white matter, eyes, and air) were first segmented from the individual T1-weighted anatomical MRI. The segmentation image of the skin tissue was subsequently smoothed to remove any residual artifact. This was carried out independently from the SimNIBS pipeline with the freely available software MIPAV by applying a spatial Gaussian filter (2 mm in each xyz direction). The creation of the head model was then completed with headreco by generating a tetrahedral mesh as volume conductor model. Next, in the SimNIBS GUI, simulated electrodes were placed manually on the head mesh at their precise position and with the corresponding orientation. Stimulation intensities were selected for anodal and cathodal electrodes and the simulation based on the finite element method (FEM) was initiated. The vector norm of the electric field (normE) was extracted and chosen as dependent variable for subsequent group-level statistical analysis. These steps were repeated separately in each active tDCS condition and in each participant.

The individual normE distribution was transformed to the fsaverage space to create a group average of the mean normE values and their standard deviation (Figure S11). This was carried out with the MATLAB scripts provided in the SimNIBS package. To assess statistical differences between stimulation conditions in the peak values and focality of the induced electric field, we extracted the 99.9 percentile value of the normE distribution, as well as the volume in which the normE values reached the 99.9 strength percentile.

### Statistical analysis

Statistical analysis was performed with the use of non-parametric permutation tests with 5000 permutations. During the baseline phase, non-parametric one-way factorial analyses with factor Stimulation (lM1, rFPC, sham) were carried out using synchronised rearrangements^66^, which are based on permutations. During learning, full $3 \times 3$ factorial analyses with factor Block (1–3 levels during learning or 1 level during baseline) and Stimulation were implemented. Effects were considered significant if $(P \le 0.05$). In the case of significant interactions, follow-up post hoc analyses using pair-wise comparisons between stimulation conditions or blocks were evaluated using pair-wise permutation tests for matched samples.

In all cases, we addressed the issue of multiple comparisons arising from the implementation of several post hoc analyses by controlling the false discovery rate (FDR) at level q = 0.05 with an adaptive two-stage linear step-up procedure^67^. Significant effects after FDR-control are reported as $P \le P_{FDR}$, and providing the explicit adapted value of $P_{FDR}$.

In addition, separately from the main factorial analyses, we performed analyses of offline (block 3) minus online (block 1) differences in IKI variability and scores in the learning phase using one-way factorial analyses with factor Stimulation (lM1, rFPC, sham). Here, post hoc analyses of pair-wise contrasts between tDCS conditions also controlled the FDR at level q = 0.05.

Throughout the manuscript, non-parametric effect sizes and corresponding confidence intervals are provided along with pair-wise permutation tests. As measure of non-parametric effect size we used the probability of superiority for dependent samples $\Delta _{dep}$, ranging 0–1^38^. Confidence intervals (CI) for $\Delta _{dep}$ were estimated with bootstrap methods^68^.

## Supplementary Information

Supplementary Information.
