# Author Correction: Modulation of neural activity in frontopolar cortex drives reward-based motor learning

**DOI:** 10.1038/s41598-021-04121-x

**Published:** 2021-12-27

**Authors:** M. Herrojo Ruiz, T. Maudrich, B. Kalloch, D. Sammler, R. Kenville, A. Villringer, B. Sehm, V. V. Nikulin

**Affiliations:** 1 Psychology Department, Goldsmiths University of London, London, UK; 2 Center for Cognition and Decision Making, National Research University Higher School of Economics, Moscow, Russian Federation; 3 Department of Neurology, Max Planck Institute for Human Cognitive and Brain Sciences, Leipzig, Germany; 4 Research Group Neurocognition of Music and Language, Max Planck Institute for Empirical Aesthetics, Frankfurt am Main, Germany; 5 Department of Neurology, University Hospital Halle (Saale), Halle, Germany

Correction to: *Scientific Reports* 10.1038/s41598-021-98571-y, published online 13 October 2021

The Acknowledgements section in the original version of this Article was incomplete.

“This research was supported by the International Engagement Fund of Goldsmiths University of London (MHR). MHR and VVN were partially supported by the National Research University Higher School of Economics (Russian Federation). We thank Dennis Maudrich for providing essential help with the experiments.”

now reads:

“This research was supported by the International Engagement Fund of Goldsmiths University of London (MHR). MHR and VVN were partially supported by the Basic Research Program of the National Research University Higher School of Economics (Russian Federation). We thank Dennis Maudrich for providing essential help with the experiments.”

The original Article has been corrected.

