# Supplementary material for: Modulation of neural activity in frontopolar cortex drives reward-based motor learning
Source: Sci Rep. 2021 Oct 13;11:20303. doi: 10.1038/s41598-021-98571-y (PMC8514446; doi:10.1038/s41598-021-98571-y)
Supplement: Supplementary file 1 — Supplementary Information. [file 41598_2021_98571_MOESM1_ESM.pdf]

Supplementary Results

Behavioural Results

General behavioural findings. During the baseline phase, general performance parameters, such as the mean tempo or keystroke velocity, did not differ as a function of the stimulation protocol ($P = 0.69$ for mean IKI; $P = 0.72$ for mean keystroke velocity). Error rates were small and did not change with stimulation ($P = 0.81$, the average error rate was $6.3[0.92]\%$). Thus, the tDCS protocols did not modulate baseline performance differently, suggesting that any differential stimulation effects on the subsequent learning phases are not confounded by baseline effects.

During the learning phase, complementing the main results (see *Results* section), we observed that the mean performance tempo changed as a function of the block number (main effect of Block; P = 0.042). This effect reflected the observed increase in performance tempo across blocks in all stimulation conditions (Figure 2D), which was related to the overall slower timing of the rewarded solution relative to their initial assumption. On average, participants played the sequences at a rate of one keystroke every 0.57 (0.036) s under lM1-tDCS, 0.58 (0.027) s under rFPC-tDCS, and 0.59 (0.031) s during sham. Lastly, error rates did not change as a function of Stimulation or learning Block ($P > 0.05$). The mean error percentage after collapsing values across stimulation conditions and blocks was small, $6.9(0.92)\%$, and resembled the rates obtained during baseline. This outcome suggests that the difficulty associated with reproducing the series of notes during learning was low and comparable to the baseline phase.

Dissociable effects of reward increases/decreases on task-related variability. We formally assessed task-related behavioural changes on a trial-by-trial basis as a function of the *history* of rewards using a computational model of the task. The results are presented in the main manuscript. As a sanity check, however, we assessed whether our task was able to capture the well known effects of reward changes on behavioural variability using a non-Bayesian analysis [1, 2]. This was assessed during sham stimulation.

Specifically, following [1], we quantified trial-to-trial behavioural exploration (unsigned changes) as a function of a drop or increase in reward on the previous trial. Here, the trialwise behavioural measure was the cv within the trial across keystroke positions, labelled cvIKI. A trial in which the participant performed the sequence with increased variation between successive IKI values would be associated with larger cvIKI, whereas an isochronous performance would lead to a cvIKI = 0. This trialwise measure was positively correlated with the observed scores (see Results), suggesting that participants modified this index as they adapted to the reward feedback.

Following [1], we represented the change from trial n to $n+1$ in cvIKI (T $\equiv$ cvIKI in the following expressions) as:

$$\Delta T^{(n+1)} = T^{(n+1)} - T^{(n)} \tag{1}$$

Reward (R) change at trial n was computed as:

$$\Delta R = R^{(n)} - R^{(n-1)} \tag{2}$$

Exploration was quantified as the unsigned trial-to-trial change in cvIKI, $|\Delta T|$ (after dropping the *n+1* index for simplicity). We analysed separately $|\Delta T|$ values that followed an increase in reward from trial $n-1$ to n, ΔR^{+}, denoting a positive sign in ΔR; and those that followed a drop in reward, ΔR^{-}, indicating a negative sign in ΔR. We then fitted a folded Gaussian distribution to the corresponding histograms of each participant's data sample during sham: $p(|\Delta T||\Delta R^{-})$ and $p(|\Delta T||\Delta R^{+})$ [1]:

$$f(|\Delta T|;\mu,\sigma) = \frac{1}{\sigma\sqrt{2\pi}}\exp\left(-\frac{(-|\Delta T|-\mu)^2}{2\sigma^2}\right) + \frac{1}{\sigma\sqrt{2\pi}}\exp\left(-\frac{(|\Delta T|-\mu)^2}{2\sigma^2}\right) \tag{3}$$

Analysis of the standard deviation (σ) of the folded conditional probability distributions $p(|\Delta T||\Delta R^{+})$ and $p(|\Delta T||\Delta R^{-})$ during sham demonstrated significantly larger σ values in trials following a decrement in scores than after an increase in scores ($P = 0.022$, Figure S5). This finding supports that our task was able to capture the previously reported effect of greater exploration following decreased reward than after an increase in reward, as expected for successful learning [1, 2].

Carry-over effects. The hidden target solution for each sequence and tDCS session was a specific value of the vector norm of IKI-differences across keystrokes in the sequence: 1.9596 (e.g. a sequence with IKI values: [0.2, 1, 0.2, 1, 0,2, 1, 0.2] s would achieve a score = 100). Participants were unaware of this aspect of the task. Yet they could have implicitly maintained the same timing solution they learnt in the previous session. We mitigated carry-over effects between sessions in two different ways:

1. counterbalancing the order of the tDCS sessions and the sequence type played across participants,

2. separating the tDCS sessions by one week. We therefore used a washout period of 7 days, as in previous work [3, 4]

Both study design choices (1) and (2) fulfill the recommendations in the literature to attenuate carry-over effects [5]. We, however, additionally assessed explicitly any potential carry-over

effect of the learned pattern of IKIs from one week to the next one. In our reward-based motor learning task there are two types of potential carry-over effects:

A Participants could perform the same pattern of IKI values from one week to the next one, despite playing a different sequence with associated auditory feedback.

B Participants could play a different IKI pattern each week but maintain the learned vector-norm from the previous week (1.9596).

Figure S6 illustrates the individual participant data in the first 10 participants. It shows that participants did not maintain the same IKI profile from one week to the next one.

To assess option (B), we calculated each week the trialwise values of the vector norm of the IKI patterns (norm of the vector of differences between consecutive IKI values). The changes in the trialwise vector-norm values within and across weekly sessions (collapsing tDCS information) are illustrated in the Figure S7. We compared statistically the average vector-norm value of the last 10 trials of a weekly session and the first 10 trials of the next weekly session (contrasts: week 1 versus week 2; week 2 versus week 3). Paired permutation tests revealed that the average vector-norm values differed between the end of week 1 and the beginning of week 2 (P = 0.0020), and similarly between the end of week 2 and the beginning of week 3 (P = 0.0008). Accordingly, the IKI patterns that participants played at the end of one week and beginning of the next week differed in the vector-norm value.

Next, we looked at the phenomenon termed "savings", which has been thoroughly investigated in the visuomotor adaptation literature [6]. Savings refers to the faster learning rates observed on successive sessions. To assess savings from week to week in our paradigm, we simply calculated the increment in our dependent variable, the vector norm of IKI differences, from the first to the last 10 trials within a weekly session, and then compared this increment ($\sim$ learning rate) between week 1 and 2 and, additionally, week 1 and 3. This statistical comparison revealed no significant differences (P = 0.9820, 0.4776, respectively). A comparison between week 2 and 3 did not reveal any significant differences either (P = 0.3534).

Accordingly, participants did not modify the vector norm of IKI differences faster in successive weeks. Neither did they start a session repeating the implicitly learned optimal vector norm value of the previous session. Lastly, they did not repeat the same profile of IKI values from week to week. These control analyses argue against carry-over effects explaining the observed tDCS findings.

Supplementary Materials and Methods

Double-blind procedure in tDCS sessions

One researcher randomly assigned participants to rFPC-tDCS, lM1-tDCS or sham-tDCS using consecutive randomisation. This researcher delivered the stimulation but was not involved in the instruction of participants on the motor task or in the acquisition of the performance data. All researchers conducting the experiment were unaware of the stimulation type.

Bayesian model of behaviour

Perceptual model

The model was implemented using the HGF toolbox (www.translationalneuromodelling.org/tapas/) for MATLAB. Regarding notation, we use lower case italics to denote scalars (x), which can be further characterised by a trial superscript x^k and a subscript i denoting the level in the hierarchy x_i^k (i = 1, 2).

In the version for continuous inputs we implemented (see [7]; function tapas_hgf.m), we used the series of feedback scores as input: $u^k \equiv$ score; normalised to range 0-1. The HGF uses the series of inputs to generate belief trajectories about external states, such as the reward value of an action or a choice. Learning occurs in two hierarchically coupled levels (x_1, x_2), one for "perceptual" beliefs (x_1: the reward tendency associated with the current performance), and the phasic volatility of those beliefs (x_2). These two levels evolve as coupled Gaussian random walks, according to:

$$x_1^k \sim \mathcal{N}(x_1^{k-1}, f_1(x_2)), \tag{4}$$

and

$$x_2^k \sim \mathcal{N}(x_2^{k-1}, \exp(\omega_2)), \tag{5}$$

with x_i^{k-1} denoting the mean of the Gaussian random walk. The variance of the random walk is governed by $f_1(x_2)$ for the first level and $\exp(\omega_2)$ for the second level. Equation 4 indicates that the lower level is coupled to the higher level through its variance (inverse precision), and depends on x_2 through an exponential function

$$f_1(x_2) \equiv \exp(\kappa x_2 + \omega_1). \tag{6}$$

Model parameter κ was fixed to 1, whereas ω_1 was a free parameter that was estimated in each participant by fitting the HGF model to the experimental data (scores and responses).

At the top level, the variance on x_2 is determined by $\exp(\omega_2)$, where ω_2 is also a free paratemer to be estimated in each individual. The specific coupling between levels indicated above has the advantage of allowing simple variational inversion of the model and the derivation of one-step update equations under a mean-field approximation. This is achieved by iteratively integrating out all previous states up to the current trial k (see appendices in [7]).

Importantly, the update equations for the posterior mean at level i and for trial k, μ_i, depend on the prediction errors weighted by uncertainty σ_i (or its inverse, precision $\pi_i = 1/\sigma_i$) according to the following expression:

$$\Delta\mu_i^k = \mu_i^k - \mu_i^{k-1} \propto \frac{\hat{\pi}_{i-1}^k}{\pi_i^k}\delta_{i-1}^k. \tag{7}$$

The first term in the above expression is the change in the *expectation* μ_i^k on trial k for state x_i, relative to the *prediction* on trial $k-1$, μ_i^{k-1}. The prediction on trial $k-1$ is also denoted by the diacritical mark ˆ: $\mu_i^{k-1} = \hat{\mu}_i^k$. The difference term $\Delta\mu_i^k$ is proportional to the prediction error of the level below, δ_{i-1}^k, representing the difference between the expectation μ_{i-1}^k and the prediction $\hat{\mu}_{i-1}^k$ of the level below x_{i-1}^k. The prediction error is weighted by the ratio between the prediction of the precision of the level below, $\hat{\pi}_{i-1}^k$, and the precision on the current level, π_i^k. The product of the precision weights ratio and the prediction error constitute the precision-weighed prediction error (pwPE), termed ϵ_i. Thus, ϵ_i regulates the update of expectations on trial k: $\Delta\mu_i^k = \epsilon_i$. The pwPE expressions for level 1 and 2 share the general form of Equation 7, and detailed definitions can be found in [7, 8, 9]. Equation 7 illustrates that higher uncertainty in the current level (larger σ_i^k, smaller π_i^k in the denominator) leads to faster update of expectations; moreover, a smaller prediction of uncertainty (larger $\hat{\pi}_{i-1}^k$) of the level below also increases the update of expectations. The intuition from this expression is that the more uncertain we are about the level we're trying to estimate (current level), the more we should update that level using new information (prediction errors). On the other hand, the less certain we are about the level below (less precise information), the less that new information should contribute to our update of beliefs.

Table S1 shows our choice of prior values on the HGF parameters that were used to generate belief trajectories, similarly to [9]. Figure S8 illustrates a schematic illustrating the model structure and the relevant variables and parameters. The priors on parameters ω_1 and ω_2 were set to be relatively uninformative by choosing a broad variance of 16. As indicated above, the coupling parameter κ was fixed to 1 and so was the intial value of the belief trajectory for the second level: $\mu_2^0 = 1$.

When analysing statistically the estimated values of the perceptual model parameters, ω_1 and ω_2, we found no significant effect of Stimulation, neither for ω_1 ($P > 0.05$, one-way factorial analysis) nor for ω_2 ($P > 0.05$). On average, ω_1 was -3.5 (0.45), and ω_2 was -4.1 (0.35).

Response Model

We implemented 16 versions of the HGF with alternative response models that explained different performance measures as a function of relevant HGF quantities. A similar approach was used in [9]. The 16 models fall into two families of related models, with each family being associated with a different performance measure: (i) The trialwise coefficient of variation of successive IKI values (cv of IKI values across sequence positions on one trial; termed cvIKI, dimensionless; N = 8 models); (ii) the trialwise mean performance tempo (mIKI, in ms, similarly to [10]; N = 8 models). Note that cvIKI was associated with the reward function, as larger differences between adjacent IKI values, which are related to a larger cvIKI, contributed towards higher reward. In fact, in each participant and session cvIKI was positively correlated with the scores (nonparametric Spearman ρ, median = 0.70, mean = 0. 68; significant in all instances). Variable cvIKI was therefore a strong candidate for the response model. Additionally, we considered the trialwise mean performance tempo to assess whether participants speeded or slowed down in each trial as a function of the observed score.

We were particularly interested in relating HGF computational quantities on the previous trial $k-1$ to changes in performance on the current trial. Therefore, as response function we chose the difference (Δ) between the performance measure in trial $k-1$ to k:

- $\Delta\mathrm{cvIKI}^{\mathrm{k}} = \mathrm{cvIKI}^{\mathrm{k}} - \mathrm{cvIKI}^{\mathrm{k}-1}$
- $\Delta\mathrm{mIKI}^{\mathrm{k}} = \mathrm{mIKI}^{\mathrm{k}} - \mathrm{mIKI}^{\mathrm{k}-1}$

And the absolute value, as a measure of unsigned changes, reflecting exploration:

- $\log(|\Delta\ \mathrm{cvIKI}^{k}|) = \log(|\mathrm{cvIKI}^{k}$ - $\mathrm{cvIKI}^{k-1})|)$
- $\log(|\Delta\ \mathrm{mIKI}^{k}|) = \log(|\mathrm{mIKI}^{k}$ - $\mathrm{mIKI}^{k-1})|)$

Note that when assessing unsigned changes we took the logarithm to transform a bounded variable (absolute value) into an unbounded variable, more suitable as dependent variable for a multiple regression (response) model.

Next, for each performance measure listed above (N = 4), the corresponding response model explained that variable as a function of different combinations of relevant HGF variables (mean of the posterior distriuton of beliefs and pwPE on reward tendency and volatility, M = 4). This

resulted in 16 different response models (N*M). See details in the main manuscript, including the winning perceptual + response model combination.

References

[1] Pekny SE, Izawa J, Shadmehr R (2015) Reward-dependent modulation of movement variability. *Journal of Neuroscience* 35(9):4015–4024.

[2] Van Mastrigt NM, Smeets JB, Van Der Kooij K (2020) Quantifying exploration in reward-based motor learning. *Plos one* 15(4):e0226789.

[3] Nitsche MA, et al. (2003) Facilitation of implicit motor learning by weak transcranial direct current stimulation of the primary motor cortex in the human. *Journal of cognitive neuroscience* 15(4):619–626.

[4] Pavlova E, Kuo MF, Nitsche MA, Borg J (2014) Transcranial direct current stimulation of the premotor cortex: effects on hand dexterity. *Brain research* 1576:52–62.

[5] Biabani M, Farrell M, Zoghi M, Egan G, Jaberzadeh S (2018) Crossover design in transcranial direct current stimulation studies on motor learning: potential pitfalls and difficulties in interpretation of findings. *Reviews in the Neurosciences* 29(4):463–473.

[6] Krakauer JW (2009) Motor learning and consolidation: the case of visuomotor rotation. *Progress in motor control* pp. 405–421.

[7] Mathys CD, et al. (2014) Uncertainty in perception and the hierarchical gaussian filter. *Frontiers in human neuroscience* 8:825.

[8] Mathys C, Daunizeau J, Friston KJ, Stephan KE (2011) A bayesian foundation for individual learning under uncertainty. *Frontiers in human neuroscience* 5:39.

[9] Sporn S, Hein T, Ruiz MH (2020) Alterations in the amplitude and burst rate of beta oscillations impair reward-dependent motor learning in anxiety. *Elife* 9:e50654.

[10] Marshall L, et al. (2016) Pharmacological fingerprints of contextual uncertainty. *PLoS biology* 14(11):e1002575.

[11] Thielscher A, Antunes A, Saturnino GB (2015) Field modeling for transcranial magnetic stimulation: a useful tool to understand the physiological effects of tms? in *2015 37th annual international conference of the IEEE engineering in medicine and biology society (EMBC)*. (IEEE), pp. 222–225.

[12] Windhoff M, Opitz A, Thielscher A (2013) Electric field calculations in brain stimulation based on finite elements: an optimized processing pipeline for the generation and usage of accurate individual head models. *Human brain mapping* 34(4):923–935.

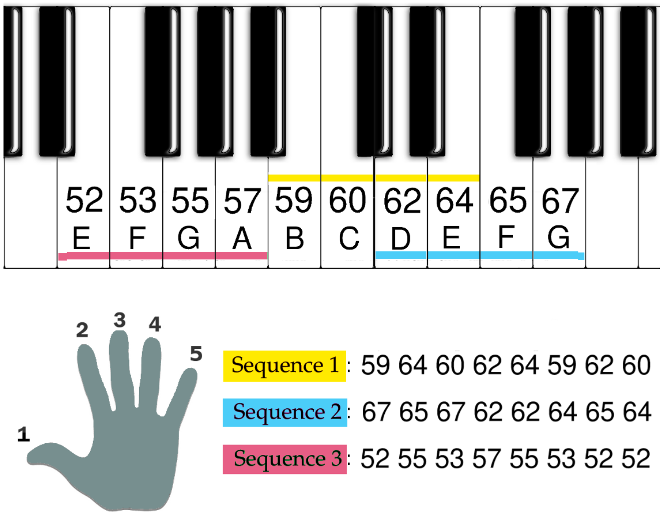

Figure S1: **Stimulus materials**. Top: The pitch content of the sequences being used for the reward-based learning blocks is displayed on the keyboard. Bottom: Sequences 1,2,3 consisted of different combinations of a set of four pitch values from four neighbouring white keys on the piano. During the preceding baseline phase, participants had to press those same keys but in a successive order (four notes upwards + same four notes downwards) regularly at a self-paced tempo and with their index to little fingers (digits 2-5 shown in the figure).

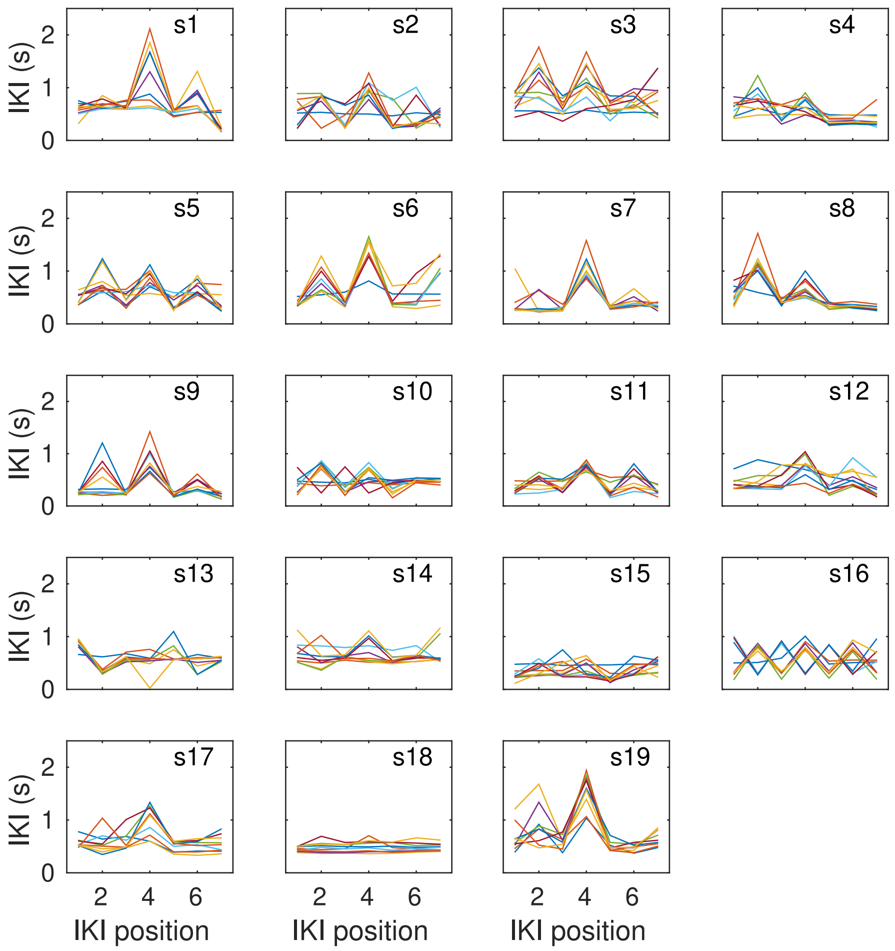

Figure S2: **Illustration of timing performance for sequence 3 during the first 10 trials in each individual participant.** The x-axis shows the position of the inter-keystroke interval (IKI; sequence 3: eight notes, corresponding to seven IKIs). The y-axis denotes the IKI value in s. Timing patterns for participants 1-19 are illustrated from left to right, top to bottom. The plots illustrate that during the first 10 trials of performance of sequence 3, participants explored similar timing solutions that were close-by in the movement space (same shape of IKI series) but also explored timing solutions that were far apart in the movement space (different shapes of IKI series across neighboring keystrokes).

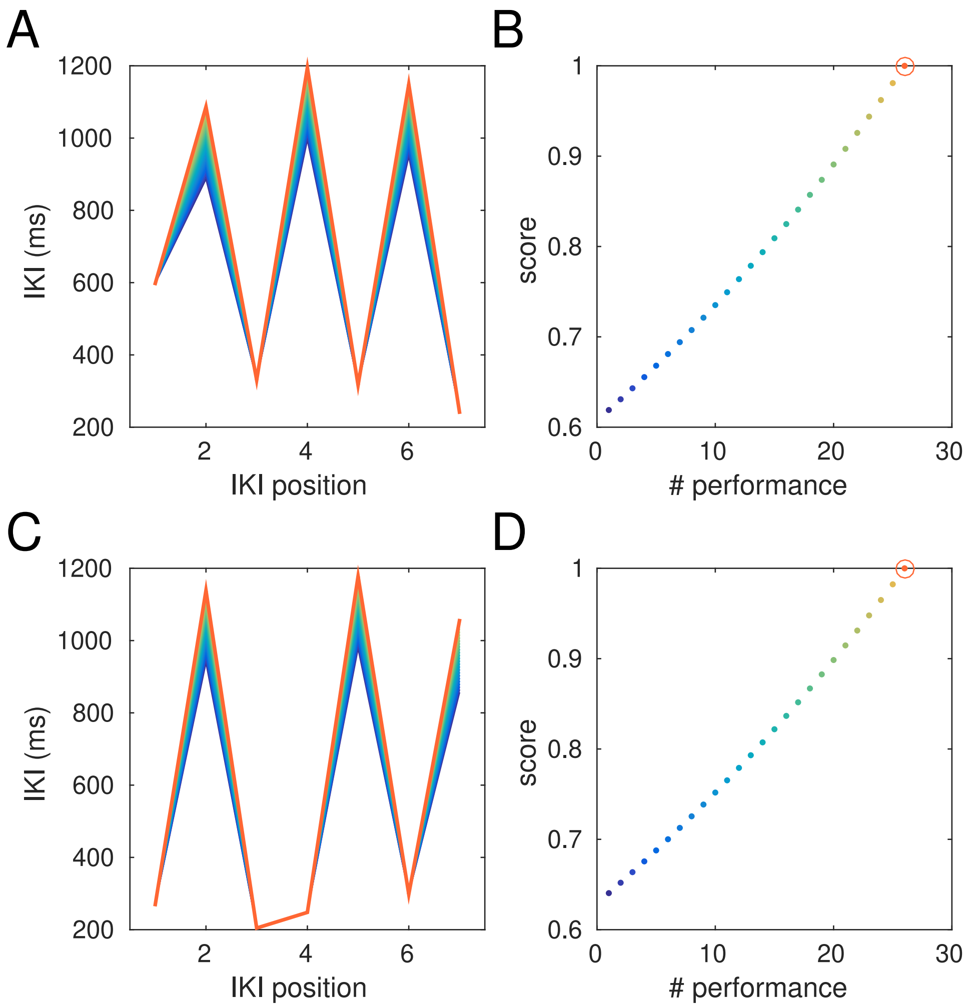

Figure S3: **Illustration of timing solutions in the movement space and their associated reward. A.** Simulated examples of trialwise timing patterns produced by an agent. The x-axis indicates the position of the inter-keystroke interval (IKI). The pitch content of our stimulus materials had eight notes, which corresponds with seven IKIs. The y-axis denotes the IKI value in ms. Different trial performances are denoted by a different color. In our task, timing patterns close-by in the movement space would obtain different rewards, as shown in panel (B). The orange curve corresponds with a maximum reward of 1 (score 100). **(B)** Reward values (normalised to 0-1) associated with each performance shown in panel (A). The color code indicates the mapping between timing pattern and reward. A maximum score of 100 is shown here as a reward value of 1 and is denoted by the orange circle. **(C)** Same as (A) but for a set of timing patterns that lie far away in the movement space from the patterns shown in (A). Comparison of the two timing solutions represented by the orange lines in (A) and (C) illustrates how in our task timing patterns that were far away in the movement space could receive the same reward (= 1 here). **(D)** Same as (B) but corresponding with the timing solutions illustrated in (C).

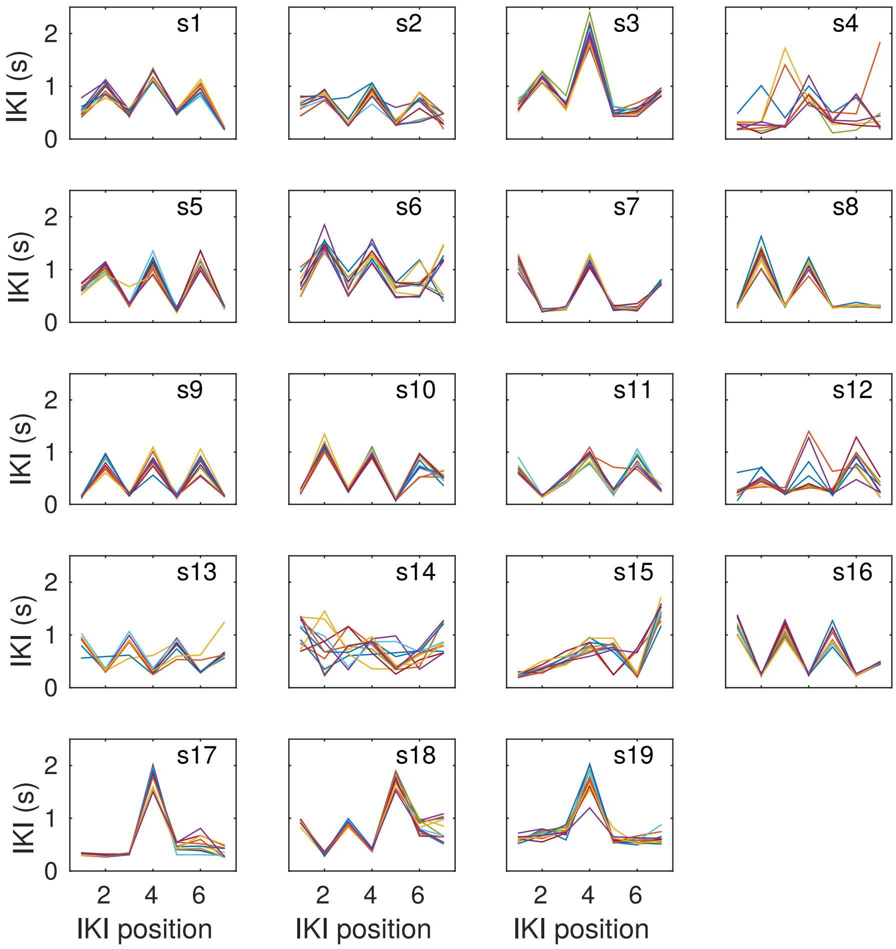

Figure S4: **Illustration of timing performance for sequence 3 during the last 10 trials in each individual participant.** Same as Figure S2 but during the last 10 trials of the experimental session corresponding with performance of sequence 3 (total number of trials: 90). This figure illustrates the more consistent exploitation of the timing pattern inferred to be rewarding. In a minority of the cases, however, exploration of different timing solutions was manifest even at the end of the experimental session (subjects 4 and 14).

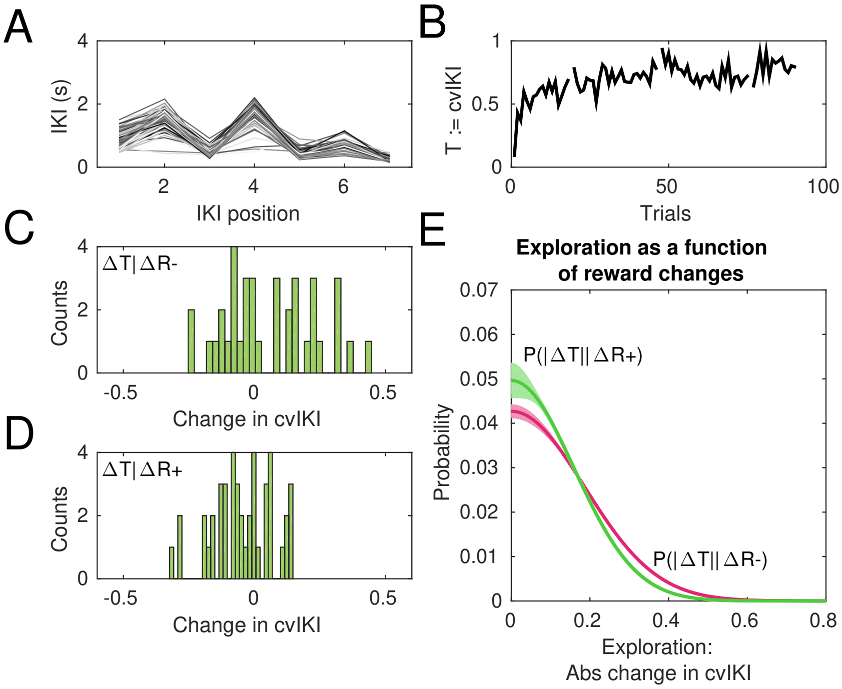

Figure S5: **Trial-to-trial exploration**. **(A)** Timing performance for sequence1 across all 90 trials in one representative participant. The x-axis shows the position of the inter-keystroke interval (IKI; eight notes, corresponding to seven IKIs). The y-axis denotes the IKI value in seconds. **(B)** The trialwise coefficient of variation of IKI intervals across keystroke positions, cvIKI (labeled T for simplicity), increased as participants approached the hidden timing pattern (and received increasingly higher scores). Representative cvIKI trace in one participant, corresponding to timing performance shown in (A). **(C-D)** Histogram distributions for data samples $\Delta T|\Delta R^-$ and $\Delta T|\Delta R^+$, reflecting signed shifts in cvIKI following changes in reward (R^- and R^+) in one representative participant. **(G)** We assessed statistically how exploration changed as a function of increases or decreases in reward during sham. Absolute changes in cvIKI—our index of trial-to-trial exploration—following decrements in reward were represented by a broader distribution (larger standard deviation: magenta distribution) than following an increase in reward ($P = 0.022$).

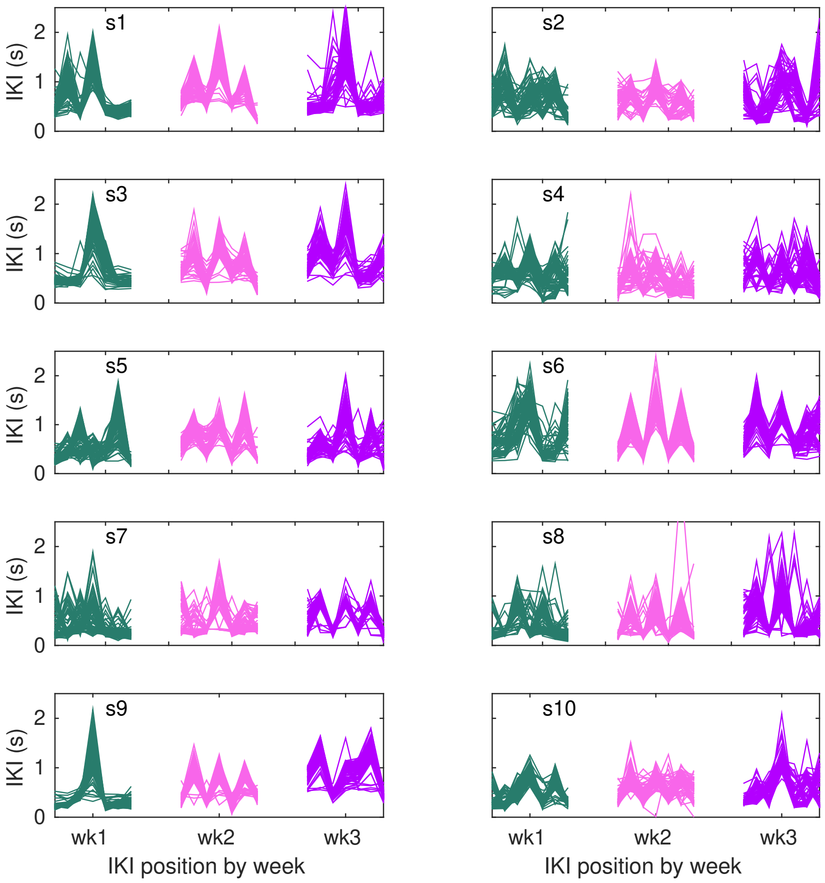

Figure S6: **IKI patterns associated with performance on different weeks**.

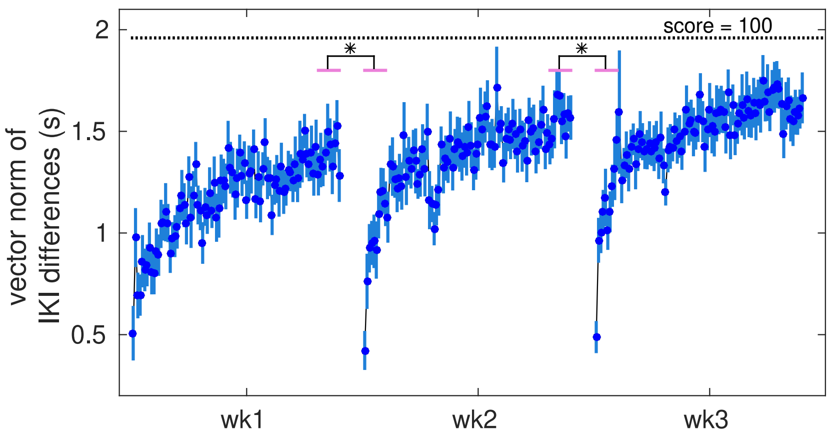

Figure S7: **Savings and carry-over effects**. The figure illustrates the trialwise values of the vector norm of the IKI differences (differences between IKI values in seconds across keystroke positions in one trial) during week 1, 2 and 3. The horizontal dashed line represents the target vector norm leading to a 100 score. A statistical comparison between the average vector-norm of the last 10 trials of a weekly session and the first 10 trials of the next weekly session (denoted by the pink lines) demonstrated significant differences for the following contrasts: week 1 versus week 2; week 2 versus week 3 (P = 0.0020 and 0.0008, respectively; significant after FDR control).

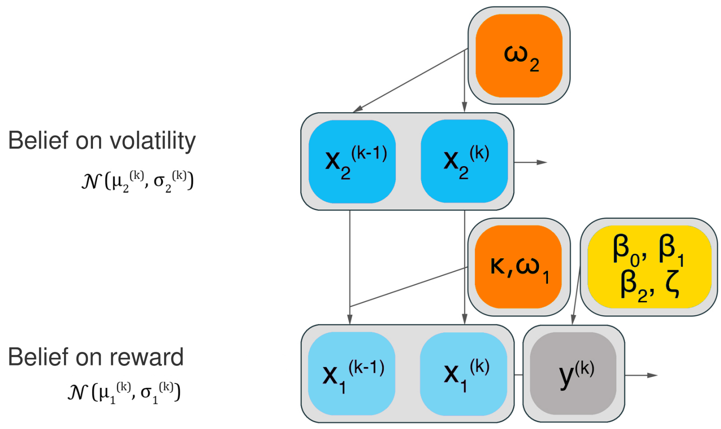

Figure S8: **Computational model**. Two-level Hierarchical Gaussian Filter for continuous inputs. **(A)** Schematic of the two-level HGF, which models how an agent infers a hidden state in the environment (a random variable), x_1, as well as its rate of change over time (x_2, environmental volatility). Beliefs (μ_1, μ_2) about those hierarchically-related hidden states (x_1, x_2) at trial k are updated with the input scores for that trial via prediction errors (PEs). The states x_1 and x_2 are continuous variables evolving as coupled Gaussian random walks, where the step size (variance) of the random walk depends on a set of parameters (shown in orange boxes). The lowest level is coupled to the level above through the variance. The response model generates the most probable response, y, according to the current beliefs about the lower state, x_1, and is modulated by the response model parameters (yellow box).

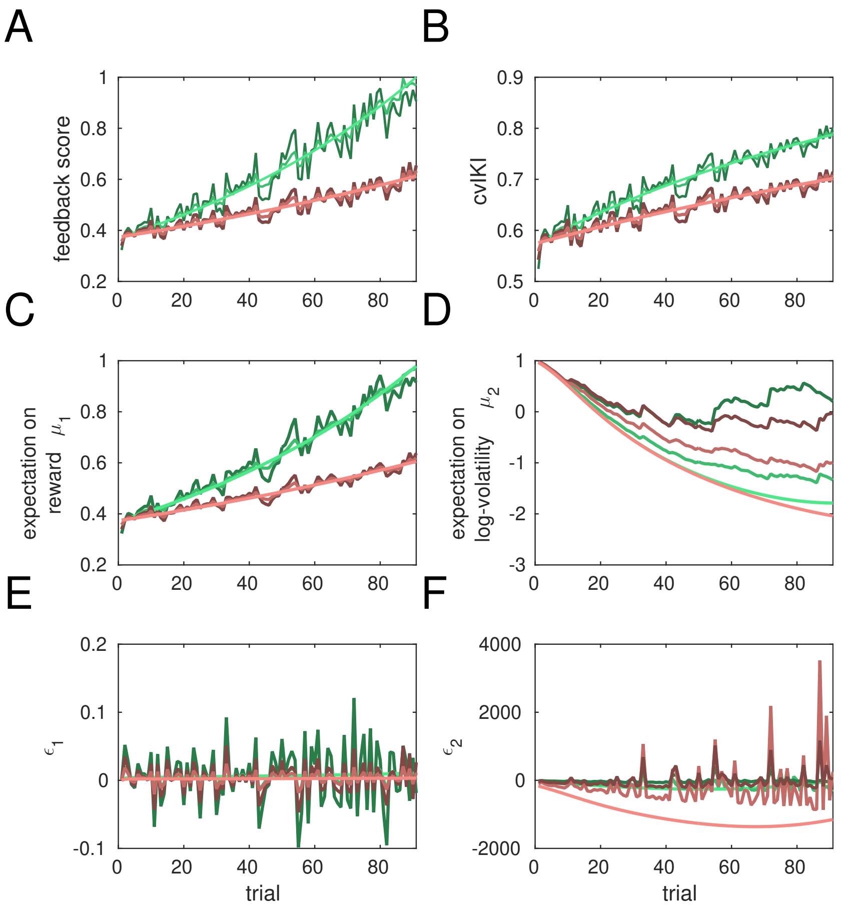

Figure S9: **Trial-by-trial belief trajectories for simulated performances.** Belief trajectories were generated using prior values on the HGF parameters as shown in **Table S1** and based on [9]. We simulated performances in six agents by changing the trial-to-trial difference in IKI values across keystroke positions, thus leading to different trajectories of cvIKI (**B**) and feedback scores (**A**). We started with a pattern of IKI values of [0.2, 0.6, 0.2, 0.6, 0.2, 0.6, 0.2] s and iteratively prolonged the inter-keystroke interval at positions 2,4,6, thereby increasing the temporal difference between IKI values, the vector norm of the total IKI pattern and the cvIKI value across keystroke positions within the trial. In the panels, steeper and shallower slopes of change across trials in cvIKI and associated feedback scores are denoted by green and pink colored lines, respectively; lighter colors denote smoother trial-by-trial transitions in cvIKI values. Darker colors indicate noisier trial-by-trial changes in this measure, representing an agent with a more variable behavioural strategy every trial. **C-D**. Expectation on reward and log-volatility. **E-F**. Precision-weighted prediction error on reward, ϵ_1, and volatility, ϵ_2. A steeper slope of change in feedback scores and cvIKI was associated with a steeper rate of change in μ_1, driven by large fluctuations in ϵ_1. Conversely, it was linked to ϵ_2 values close to 0 and a higher expectation on log-volatility μ_2 (due to a smaller attenuation from the prior value at 1). For a fixed slope, increasing levels of noise in the cvIKI and score trajectories also contributed to higher expectation on volatility and reduced ϵ_2. Thus, agents either (i) introducing more fluctuations in behaviour from trial to trial or (ii) observing a faster rise in scores had a higher expectation on volatility, which decreased less over time.

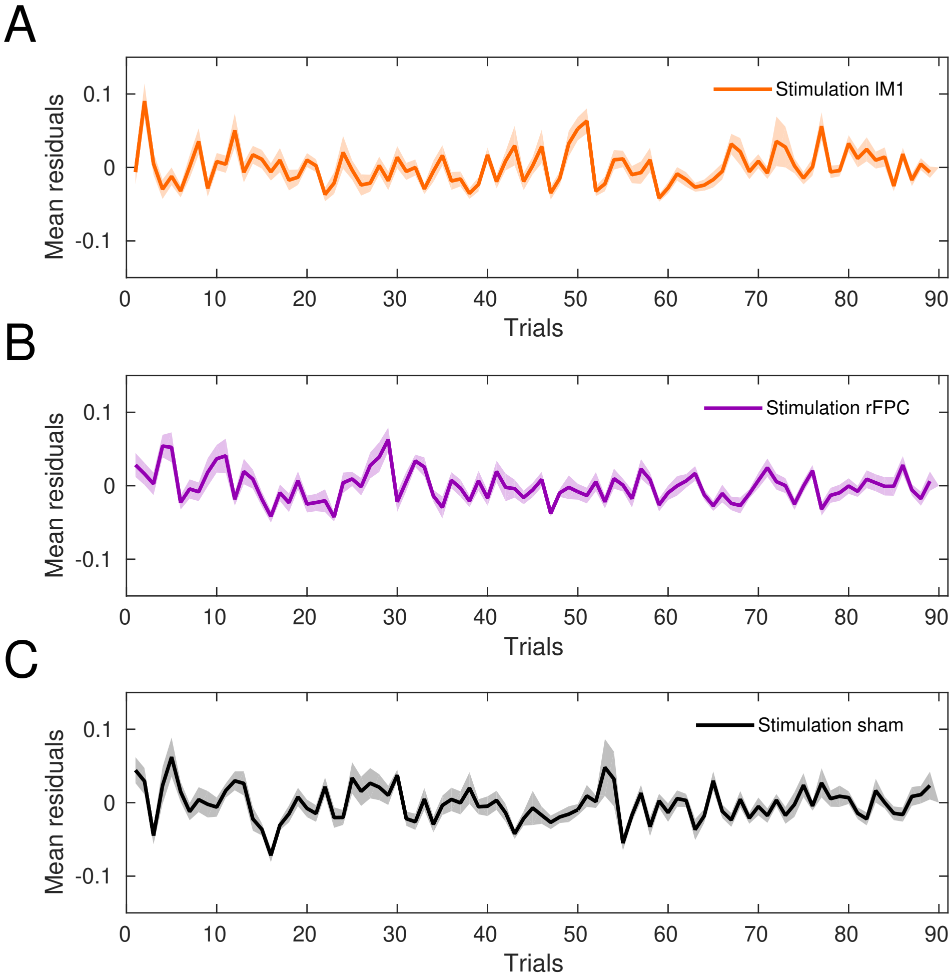

Figure S10: **Grand-average trialwise residuals resulting from the difference between the observed responses and the responses predicted by the HGF**. **(A-C)** The trialwise residuals in each tDCS stimulation condition are shown separately in panels A-C as mean and SEM (shaded areas). The winning model used as response variable the logatithm of the trial-to-trial unsigned change in cvIKI, log(|Δ cvIKI|), reflecting trialwise exploration of IKI variation across keystrope positions. There were no systematic differences in the model fits across stimulation conditions ($P > 0.05$, one-way non-parametric factorial analysis).

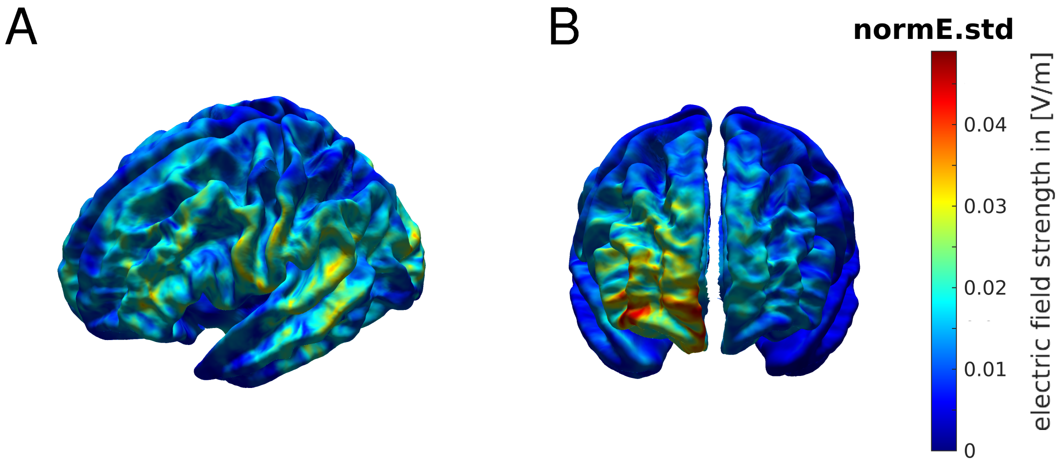

Figure S11: **Variability of electric field distribution for anodal lM1-tDCS and rFPC-tDCS** Electric field distribution for anodal tDCS protocols (left: lM1; right: rFPC), derived from FEM calculations using SimNIBS. Variability of the electric field strength (standard deviation of normE) averaged across all participants using the fsaverage-transformed surface. Figure created with the freely available SimNIBS 2.1 software [11, 12], https://simnibs.github.io/simnibs.

Table S1: **Priors on the parameters and initial values of HGF perceptual model for continuous inputs.** Means and variances of the priors on perceptual and response parameters and initial values are provided. The continuous inputs here were the trial-by-trial scores that participants received, normalised to the 0-1 range. Quantities estimated in the logarithmic space are denoted by log(). Prior mean and variance for μ_1^0, as well as the prior mean for σ_1^0, ω_1 and the precision of the input, π_u^0, were defined by the initial 20 input values. When providing prior values that depend on the first 20 input scores of each individual participant, we indicate the median across the sample of 19 participants. For the remaining quantities, the prior mean and variance were pre-defined according to the values indicated in the table.

	Prior mean	Prior variance
$\log(\kappa)$	log(1)	0
ω_1	log-variance of 1:20 input scores: -3.04	16
ω_2	-4	16
$\log(\pi_u^0)$	negative log-variance of 1:20 input scores: 3.04	4
μ_1^0	value of the first input score: 0.21	variance of 1:20 input scores: 0.05
$\log(\sigma_1^0)$	log-variance of 1:20 input scores: -3.04	1
μ_2^0	1	0
$\log(\sigma_2^0)$	log(0.1)	1
β_0	individual mean of behavioural parameter	4
β_1	0	4
β_2	0	4
$\log(\zeta)$	log(0.005)	0.1
